# G-quadruplexes in an SVA retrotransposon cause aberrant TAF1 gene expression in X-linked dystonia parkinsonism

**DOI:** 10.1093/nar/gkae797

**Published:** 2024-09-17

**Authors:** Giulia Nicoletto, Marianna Terreri, Ilaria Maurizio, Emanuela Ruggiero, Filippo M Cernilogar, Christine A Vaine, Maria Vittoria Cottini, Irina Shcherbakova, Ellen B Penney, Irene Gallina, David Monchaud, D Cristopher Bragg, Gunnar Schotta, Sara N Richter

**Affiliations:** Department of Molecular Medicine, University of Padua, via A. Gabelli 63, 35121 Padua, Italy; Department of Molecular Medicine, University of Padua, via A. Gabelli 63, 35121 Padua, Italy; Department of Molecular Medicine, University of Padua, via A. Gabelli 63, 35121 Padua, Italy; Department of Molecular Medicine, University of Padua, via A. Gabelli 63, 35121 Padua, Italy; Department of Science and Technological Innovation, University of Piemonte Orientale, Viale Teresa Michel 11, 15121, Alessandria, Italy; Molecular Biology Division, Biomedical Center, Ludwig Maximilian University of Munich, Großhaderner Strasse 9, 82152 Planegg-Martinsried, Germany; Department of Neurology, Massachusetts General Hospital, Building 149 13th Street, Charlestown, MA 02129, USA; Department of Molecular Medicine, University of Padua, via A. Gabelli 63, 35121 Padua, Italy; Molecular Biology Division, Biomedical Center, Ludwig Maximilian University of Munich, Großhaderner Strasse 9, 82152 Planegg-Martinsried, Germany; Department of Neurology, Massachusetts General Hospital, Building 149 13th Street, Charlestown, MA 02129, USA; Department of Molecular Medicine, University of Padua, via A. Gabelli 63, 35121 Padua, Italy; Institut de Chimie Moleculaire de l'Université de Bourgogne, ICMUB CNRS UMR6302, 9, Rue Alain Savary, 21078 Dijon, France; Department of Neurology, Massachusetts General Hospital, Building 149 13th Street, Charlestown, MA 02129, USA; Molecular Biology Division, Biomedical Center, Ludwig Maximilian University of Munich, Großhaderner Strasse 9, 82152 Planegg-Martinsried, Germany; Department of Molecular Medicine, University of Padua, via A. Gabelli 63, 35121 Padua, Italy; Microbiology and Virology Unit, Padua University Hospital, via Giustiniani 2, 35121 Padua, Italy

## Abstract

G-quadruplexes (G4s) are non-canonical nucleic acid structures that form in guanine (G)-rich genomic regions. X-linked dystonia parkinsonism (XDP) is an inherited neurodegenerative disease in which a SINE–VNTR–Alu (SVA) retrotransposon, characterised by amplification of a G-rich repeat, is inserted into the coding sequence of *TAF1*, a key partner of RNA polymerase II. XDP SVA alters *TAF1* expression, but the cause of this outcome in XDP remains unknown. To assess whether G4s form in XDP SVA and affect *TAF1* expression, we first characterised bioinformatically predicted XDP SVA G4s *in vitro*. We next showed that highly stable G4s can form and stop polymerase amplification at the SVA region from patient-derived fibroblasts and neural progenitor cells. Using chromatin immunoprecipitazion (ChIP) with an anti-G4 antibody coupled to sequencing or quantitative PCR, we showed that XDP SVA G4s are folded even when embedded in a chromatin context in patient-derived cells. Using the G4 ligands BRACO-19 and quarfloxin and total RNA-sequencing analysis, we showed that stabilisation of the XDP SVA G4s reduces *TAF1* transcripts downstream and around the SVA, and increases upstream transcripts, while destabilisation using the G4 unfolder PhpC increases *TAF1* transcripts. Our data indicate that G4 formation in the XDP SVA is a major cause of aberrant *TAF1* expression, opening the way for the development of strategies to unfold G4s and potentially target the disease.

## Graphical Abstract

**Graphical Abstract:**
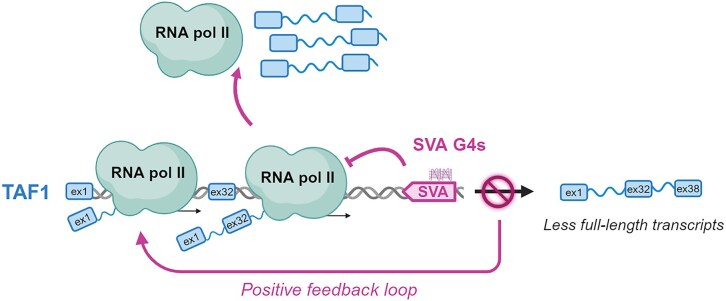

## Introduction

X-linked dystonia parkinsonism (XDP) is a neurodegenerative movement disorder endemic to the Philippines that was first reported in the literature more than 40 years ago (1). The clinical manifestations of XDP are heterogeneous and consist of both hyperkinetic and hypokinetic features that progress over time (2). This progression is directly related to neurodegeneration in specific areas of the brain, particularly atrophy of the striatum in the basal ganglia, as in the Parkinson’s disease (3). Unlike other neurological diseases where the pathogenesis is unclear, XDP has a genetic cause, with all XDP patients displaying a shared haplotype in the Xq13.1 region of the X chromosome.

Initial mapping of the XDP haplotype identified seven variants that appear to be inherited together in all patients reported to date: five disease-specific single-nucleotide changes (DSCs 1, 2, 3, 10 and 12), one 48-bp deletion (4) and a SINE–VNTR–Alu (SVA) retrotransposon antisense insertion (5). These variants are all located within and around the *TAF1* gene, which encodes the TATA-box binding protein association factor 1, essential for RNA polymerase II activity (6). DSCs 1, 2 and 3 together with the 48-bp deletion are found in the intergenic region downstream of the *TAF1* gene that has been designated as a multiple transcript system (7). DSCs 10 and 12 are located in intron 32 and intron 18 of *TAF1*, respectively (4). The SVA is found in intron 32 of the *TAF1* gene, upstream of DSC 10 (5). Subsequent genomic analyses have further refined the XDP haplotype, narrowing the region common to all probands to a 219.7-kb segment that spans *TAF1* and includes the seven original markers and six additional disease-specific variants (8).

The XDP SVA is a composite non-long terminal repeat (non-LTR) retrotransposon belonging to the SVA-F family. It is composed at the 5′ of a hexanucleotide repeat (CCCTCT)_*n*_, named hexameric domain (HEX), an Alu-like region formed by two antisense Alu fragments separated by an intervening sequence, one variable number tandem repeat (VNTR) region, a short interspersed nuclear element (SINE) region derived from the 3′ LTR of the HERV-K10 retroviral element and a poly-A signal at the 3′ end (9). The HEX is highly amplified [from 32 to 50 times (10)] in the XDP SVA compared with the four to eight repetitions that are commonly found in other SVAs unrelated to the disease (11).

Of all XDP mutations, the SVA insertion is currently thought to be the most relevant to the XDP pathogenesis, possibly due to its effect on *TAF1* expression. XDP cell models exhibit multiple transcriptional abnormalities surrounding the exons flanking the SVA insertion, including aberrant alternative splicing, partial intron retention, decreased transcription of exons 3′ to the SVA and decreased levels of the full-length *TAF1* messenger RNA (mRNA) (8). Furthermore, excision of the SVA has been shown to rescue all of these defects and restore normal levels of *TAF1* transcript (8). Most importantly, the number of HEX repeats within the SVA is polymorphic in XDP patients and inversely correlated to the age of disease onset (10), suggesting that the XDP SVA plays a causal role in the development of XDP. However, the mechanisms by which the XDP SVA influences *TAF1* expression remain elusive.

XDP SVA is a genomic element with high guanine/cytosine GC content, with around 65% of the sequence being guanines (Gs) (12). Such G-rich DNA sequences have a high propensity to fold into non-canonical secondary structures known as G-quadruplexes (G4s). Accordingly, it has been previously suggested that G4s could form in the XDP SVA (10).

G4s are four-stranded DNA structures formed by the staking of G-quartets, which consist of four Gs in a planar array stabilised by Hoogsteen hydrogen bonds and monovalent cations, such as Na^+^ or K^+^, but Li^+^ does not stabilise due to its smaller size (13). At least two G-quartets are necessary to form stable G4s (14). Depending on mutual strand orientation, loop length and sequence, G4s adopt parallel, antiparallel or hybrid conformations (15). G4s have been visualised in cells (16) and are non-randomly distributed throughout the genome, with high enrichment at gene promoters and enhancers of active genes (17), where they are thought to regulate transcription by different mechanisms (18). Several G4-binding proteins, such as SP1, are transcription factors and have been shown to bind to promoter G4s and increase gene transcription (19,20). Furthermore, protein-independent mechanisms of G4-mediated enhancement of transcription have been reported, involving the formation of R-loops at the C-rich strand opposite the G4 (21,22).

G4s have also been more recently investigated as a source of DNA replication-dependent genomic instability, being either DNA replication- or transcription-dependent instability (23). In fact, stable G4s can block replication fork progression (24) leading to DNA damage. In this context, G4 sites have shown mutagenic potential, particularly after loss of the enzymes dedicated to physiological G4 processing (25). G4s may also constitute a barrier during transcription: DNA G4s, particularly when located within actively transcribed genes, could halt the transcription process if G4s are not resolved (26). In addition, stalling of the RNA polymerase complex may facilitate the recruitment of splicing factors, leading to alternatively spliced forms of the transcribed gene (27).

Here, we set out to investigate the formation and effect of XDP SVA G4s on *TAF1* transcription. *In vitro* data have suggested formation of G4 structures within SVA retrotransposons (28); however to date, there are no data to support G4 formation at the XDP SVA in cells. We first predicted and characterised XDP SVA G4s *in vitro*; we next demonstrated the actual folding of G4s at the HEX repeat region in both human fibroblasts (hFib) and neural progenitor cells (NPCs) derived from XDP-affected patients. Upon treatment of XDP cells with G4 ligands and unfolders, we observed modulation of *TAF1* mRNA levels, providing the first evidence that *TAF1* transcription in XDP is modified by G4s.

## Materials and methods

### Primers and oligonucleotides

Desalted primers and oligonucleotides were purchased from Sigma–Aldrich (Milan, Italy). A detailed list of primer names and sequences can be found in Supplementary Tables S3–S6.

### G4 prediction

The presence of putative G4s within the XPD SVA was assessed using the QGRS Mapper; the QGRS tool was used online at https://bioinformatics.ramapo.edu/QGRS/ with the following parameters: Max Length:30 - MinG-Group Size:3 - Loop Size:from 0 to 10. The XDP SVA sequence was obtained from NCBI GenBank AB191243.135.

### Circular dichroism

DNA oligonucleotides were diluted to a final concentration of 2 μM in lithium cacodylate buffer (10 mM, pH 7.4, KCl 10–100 mM). VNTR sequences were tested at 10 mM KCl because of their high stability, whereas the hexameric sequence was tested at 100 mM KCl, which more closely mimics the K^+^ concentration in the cell nucleus. All samples were denatured at 95°C for 5 min and gradually cooled to room temperature to allow G4 folding. Where indicated, compounds, namely quarfloxin (Q; MedChemExpress, #HY-14776) and BRACO-19 (B19; ENDOTHERM, Saarbruecken, Germany), were added to oligonucleotides 4 h after folding, at 4-molar excess. PhpC was added at 10-molar excess. Circular dichroism (CD) spectra were recorded on a Chirascan-Plus (Applied Photophysics, Leatherhead, UK) equipped with a Peltier temperature controller using a quartz cell of 5 mm optical path length, over a wavelength range of 230–320 nm. For the determination of *T*_m_, spectra were recorded over a temperature range of 20–90°C, with temperature increase of 5°C. The reported spectra are baseline-corrected for signal contributions due to the buffer. Observed ellipticities were converted to mean residue ellipticity (*θ*) = deg × cm^2^ × dmol^−1^ (mol ellip). *T*_m_ values were calculated according to the van’t Hoff equation, applied for a two-state transition from a folded to an unfolded state, assuming that the heat capacity of the folded and unfolded states is equal. All oligonucleotides were tested at least twice in independent experiments.

### DMS footprinting

The DNA substrate of interest was purified by polyacrylamide gel electrophoresis before use, 5′-end-labelled with [γ^32^P] ATP by T4 polynucleotide kinase and purified using MicroSpin G-25 columns (GE Healthcare Europe, Milan, Italy). Radiolabelled oligonucleotides were then resuspended in lithium cacodylate buffer 10 mM at pH 5.4 and 7.4, with or without KCl 100 mM, heat denatured and folded overnight. Sample solutions were then treated with dimethyl sulfate (DMS; 0.5% in ethanol) for 5 min at room temperature and stopped by addition of 10% glycerol and β-mercaptoethanol. Samples were loaded onto a 15% native polyacrylamide gel and run until the desired resolution was obtained. DNA bands were localised via autoradiography, excised and eluted overnight. The supernatants were recovered, ethanol-precipitated and treated with piperidine 10% (v/v) for 30 min at 90°C. Samples were dried in a speed-vac, washed with water, dried again and resuspended in formamide gel loading buffer. Reaction products were analysed on 20% denaturing polyacrylamide gels, visualised by phosphorimaging analysis and quantified by ImageQuant TL software (GE Healthcare Europe, Milan, Italy).

### *Taq* polymerase stop assay

The 5′-FAM-labelled DNA primer (final concentration 72 nM) was annealed to the template (final concentration 36 nM) in lithium cacodylate buffer (10 mM, pH 7.4) in the presence or absence of 100 mM KCl, by heating at 95°C for 5 min and gradually cooling to room temperature to allow both primer annealing and G4 folding, and incubated overnight. Where indicated, the G4 ligands B19 and Q were added 4 h post-annealing, at the final concentrations of 1 μM and 200 nM, respectively. Primer extension was achieved by adding 2 U/reaction of AmpliTaq Gold DNA Polymerase (Applied Biosystems, Carlsbad, CA, USA) at 42°C for 30 min. Reactions were stopped by sodium acetate precipitation, and primer extension products were separated on a 16% denaturing gel, and finally visualised by fluorescence gel scanner (Typhoon FLA 9000) and quantified by ImageQuant TL software (GE Healthcare Europe, Milan, Italy).

### Cell culture

Human patient XDP and control fibroblasts (fNMC, fXDP and fWT, respectively) derived by the Collaborative Center for X-Linked Dystonia-Parkinsonism (Massachusetts General Hospital, Boston, MA) were obtained from the NINDS Human Cell and Data Repository (RUCDR Infinite Biologics, Piscataway Township, NJ). Phenotypic characterisation of donors and derivation of fibroblast lines have been previously reported (29). Fibroblasts were maintained in Dulbecco’s modified Eagle’s medium (DMEM) high glucose (Gibco, #11965092) with 15% Fetal Bovine Serum (FBS) (Gibco, #10270106), 1× non-essential amino acids (Sigma–Aldrich, #M7145) and 1× penicillin–streptomycin (Gibco, #15140–122). Some experiments were performed on human XDP and control NPCs (nXDP and nWT, respectively) that were differentiated from induced pluripotent stem cell (iPSC) lines reprogrammed from patient and control fibroblasts. Cellular reprogramming, iPSC characterisation and differentiation of NPCs have been previously described (8,29,30). NPCs were cultured on lactose dehydrogenase elevating virus (LDEV)-Free human embryonic stem cell (hESC)-Qualified Geltrex (Thermo Fisher, #A143302)-coated plates in neural progenitor medium made with DMEM F12 (Gibco, #11320033) supplemented with 2% B27 (Gibco, #17504044), 1% penicillin/streptomycin (Gibco, #15140-122), 20 ng/ml Epidermal growth factor (EGF) (Peprotech, #AF-100-15-100UG), 20 ng/ml Fibroblast Growth Factor-basic (bFGF) (Millipore, #GF003) and 5 μg/ml heparin (Sigma–Aldrich, #H3149-100KU). All cell lines used in this study are summarised in Table 1.

**Table 1.** Cell lines used in this study

Patient	XDP onset age	Biopsy age	Siblings	#ID	hFib	NPCs
Healthy ctrl (WT)		18	Son of XDP affected	34430	fWT	
XDP at risk (NMC)		7	Nephew of XDP affected	35616	fNMC	
XDP affected (XDP)	32	35	Proband	32517	fXDP	nXDP #02
XDP affected (XDP)	unknown	45	Proband	35833		nXDP #01
Healthy ctrl (WT)		34	Son of XDP affected	33114		nWT #01
Healthy ctrl (WT)		34	Son of XDP affected	33362		nWT #02

For each cell line, the donor, the age at XDP disease onset and the age at biopsy are reported. The ‘siblings’ column indicates the relationship to the XDP-affected cell line (proband). The original ID (#ID) and the acronyms used in this study for the hFib and NPCs are shown.

### PCR and nested PCR stop assay

Genomic DNA (gDNA) was extracted from 1.5 × 10^6^ cells using the GeneJET Genomic DNA Purification Kit (Thermo Fisher, #K0721) according to the manufacturer’s instructions. One hundred fifty nanograms of gDNA was used for polymerase chain reaction (PCR) XDP SVA amplification using PrimeSTAR GXL enzyme (Takara, #R050A) as previously reported (31). For the SVA PCR stop assay, 150 ng of gDNA was folded into G4: KCl (0–100 mM) was added and the mixture was heated at 95°C for 5 min, and then the tubes were left at room temperature (RT) O/N. The next day, the specified G4 ligand was added to the mixture and allowed to equilibrate for 6 h at RT in the dark. Samples were then used for SVA PCR amplification with the same protocol described earlier. A total of 10 μl of PCR samples was loaded onto a 0.8% agarose gel with GelRed (Millipore, #SCT122) and run at 70 V for 1 h. For nested PCR, we gel-purified the SVA amplicon by SpinNAker Gel&PCR DNA Purification Kit (Euroclone, #EMR602050). A total of 1 ng of purified SVA was used as a template for PCR using different parameters for each domain. We designed domain-specific primers with Primer3plus (32). A complete list of primers and PCR cycling protocols used for each domain is listed in Supplementary Table S4. Moreover, 10 μl of PCR samples was loaded on agarose gel 1.2% with GelRed (Millipore, #SCT122) and run at 70 V for 1 h. Gels were visualised with a Typhoon gel scanner. Bands were quantified using ImageQuant TL software (GE Healthcare Europe, Milan, Italy).

### G4-ChIP-qPCR/seq

The G4-ChIP protocol was adapted from a previously reported protocol (33). For nuclei isolation and shearing buffers, we used the buffer composition reported by Schmidt *et al.* (34). Here, 2 × 10^6^ of fixed hFib nuclei were sheared at 35×(30 s ON/60 s OFF), 3 × 10^6^ NPCs at 40×(30 s ON/60 s OFF) in 300 μl lysis buffer on a Bioruptor PLUS (Dianogenode, Belgium). Then, 1 μg of sheared chromatin was incubated with 0.5 μg BG4 antibody (Merck, #MABE917) for 1 h at 16°C. After binding, anti-FLAG^®^ M2 beads (Merck, # M8823) were added and incubated at 16°C for 1 h. Then, beads with immunoprecipitated chromatin were washed five times with ice-cold wash buffer and DNA was de-cross-linked and purified as indicated elsewhere (19). The eluted DNA was subjected to qPCR using TaqMan™ Universal PCR Master Mix (Applied Biosystems, #4304437) with specific primers and Taqman probe for each region of interest (Supplementary Table S5), or library preparation using the ThruPLEX DNA-Seq Kit (Takara, #R400674) with dual indexes. Libraries were purified with AMPure XP beads (Beckman Coulter, #20044300); quality check was performed by qPCR to check specific enrichment for G4-positive regions and by Bioanalyzer (Agilent, USA) using High Sensitivity DNA Kit (Agilent, # 5067-4626) to check library size distribution and adaptor contamination. Libraries were sequenced 50 bp paired-end on a NextSeq 1000 Illumina platform by LAFUGA (Genecenter, Munich).

All bioinformatic analyses were performed on a cluster at the Biomedical Center (LMU, Munich). Reads were quality checked with FASTQC and aligned to hg38 reference genome with bowtie2 (35). For XDP patient, we generated a custom genome reference inserting the SVA in the hg38 reference genome using reform (https://github.com/gencorefacility/reform). The SVA sequence was retrieved from NCBI #AB191243. Alignments were sorted, indexed, removed of duplicates and converted to Binary Alignment Map (BAM) files using SAMtools (36). BigWig files were generated with deepTools bamCoverage (37). Repeat analyses were performed using Homer (38). Profile plots were performed using deepTools suite (37). Graphs were obtained using ggplot2 and on R (version 4.2).

### Cytotoxicity assay

Cells were seeded with 80% confluence in a 96-well plate. The next day, cells were treated with increasing concentrations of G4 ligands and, after 24 h of incubation, ATPlite Luminescence Assay System (Perkin Elmer, # 6016943) was applied using manufacturer’s instructions. Luminescence was acquired by a Victor X2 microplate reader (Perkin Elmer, Waltham, MA, USA).

### G4 ligand treatment using Realtime-qPCR and RNA-seq

hFib were seeded in a 6-well plate and NPCs were seeded in a 24-well plate to obtain 70% confluence. The next day, cells were treated with B19, Q or PhpC. At 24 h post-treatment, RNA was extracted with Total RNA Purification Plus Micro Kit (Norgen Biotek, #NR48500). RNA concentration was checked using Qubit (Thermo Fisher Scientific, USA). For Realtime-qPCR (RT-qPCR), 200 ng of extracted RNA was retrotranscribed with SuperScript™ III Reverse Transcriptase (Thermo Fisher Scientific, #18080044) using random hexamers. Complementary DNA was diluted and used for real-time qPCR with TaqMan™ Universal PCR Master Mix (Applied Biosystems, # 4304437) on a QuantStudio™ 3 Real-Time PCR System (Applied Biosystems, USA). Row Ct data were normalised using actin as a housekeeping gene and the −ΔΔCt method for normalisation. Data were plotted using GraphPad (Prism 2020) and statistical analysis was performed using an unpaired *t*-test. For RNA-seq analyses, the quality was checked using the TapeStation System (Agilent, USA), respectively, before and after library construction. RNA-seq libraries were generated using Illumina Stranded mRNA Prep (Illumina, USA). Sequencing was performed on a NextSeq500 Illumina instrument to produce at least 35 million reads (75 bp PE) per sample. The experiment was performed in three independent biological replicates. Reads were quality checked with FASTQC and aligned to hg38 or XDP custom genome with STAR (39). Alignments were sorted and indexed with SAMtools (36). BigWig files were generated with deepTools bamCoverage (37). RNA-seq counts were generated with HTSeq (40). Differential expression (DE) analysis was performed with the DESeq2 R package (41). Alternative splicing analysis was performed with the Splicewiz R package (42). Graphs were obtained using ggplot2 and on R (version 4.2).

## Results

### G4 prediction at the XDP SVA

**Figure 1.**
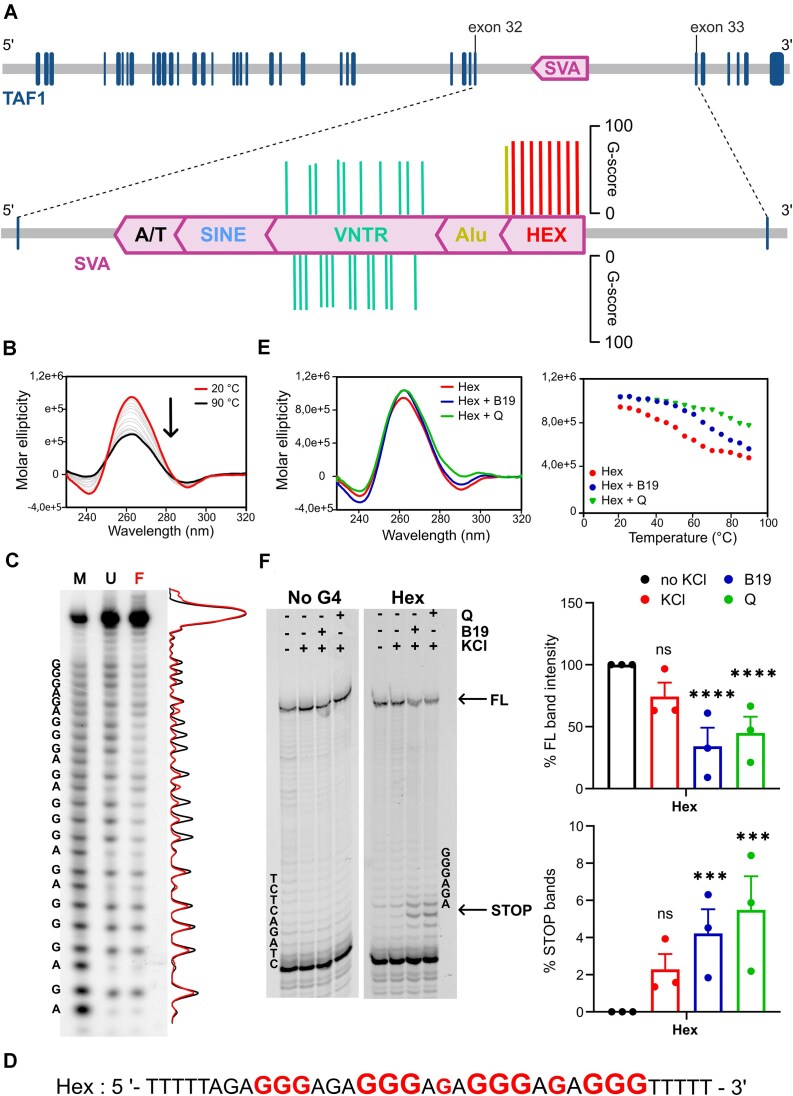
XDP SVA G4 prediction and characterisation *in vitro*. (**A**) Schematic representation of the XDP SVA retrotransposon antisense insertion within the *TAF1* gene. Each bar corresponds to a pG4 predicted by QGRS (43). Putative G4 sequences are clustered in the VNTR (green bars) and HEX (red bars) regions. (**B**) CD melting analysis of the Hex sequence. Increasing the temperature leads to loss of the G4 parallel topology, as indicated by the arrow. (**C**) DMS footprinting analysis of the folded (F) and unfolded (U) Hex sequence was performed to identify the putative Gs involved in G-tetrad formation. M stands for sequence marker for adenines and Gs obtained with the Maxam and Gilbert protocol. The black (unfolded) and red (folded) densitograms indicate the degree of protection of each G. (**D**) The Hex sequence according to the DMS footprinting output. Bases protected from DMS alkylation/cleavage are shown in red. The font size is proportional to the degree of protection. (**E**) CD analysis of the Hex G4, alone (red) or with G4 ligands, B19 (blue) and Q (green). (Left panel) CD spectra were recorded at 25°C: they represent a parallel G4 topology with a positive peak at 265 nm. Both compounds show an increase in molar ellipticity, indicating stabilisation of the G4 structure. (Right panel) Melting curves showing stabilisation of the G4 structure mediated by the two compounds. (**F**) *Taq* pol stop assay (left panel). The Hex sequence and negative control templates were treated in the absence or presence of G4-stabilising conditions (KCl and G4 ligand B19 or Q). Under the latter conditions, only the full-length product of the Hex template was reduced. At the same time, stop bands were more intense in the presence of B19 or Q, indicating the ability of the stabilized G4 structure to interfere with enzyme activity. Quantification of bands in the *Taq* pol stop assay (right panel). Full-length and stop bands were quantified from both no G4 and Hex templates in different conditions of KCl and G4 ligands. Error bars represent standard deviation of three independent experiments. Statistical analyses were performed using ANOVA test with multiple comparisons setting the condition without KCl as reference (ns = *P* ≥ 0.05, *P < 0.05, **P < 0.01, ***P < 0.001 and ****P ≤ 0.0001).

Given the GC richness of the XDP SVA, we searched for putative G4-forming sequences (pG4s) in both the sense and antisense strands using the available bioinformatic tool QGRS Mapper (43) (Figure 1A and Supplementary Table S1). Two G-rich domains, namely VNTR and HEX, presented a high pG4 content. With respect to the *TAF1* gene, at least 20 pG4s were found in the sense strand: 10 pG4s in the VNTR, 1 pG4 in the Alu, and from 9 to 18 pG4s in the HEX, depending on the length of the expansion. In the antisense strand, 13 pG4s were all in the VNTR domain (Supplementary Table S1). The pG4s in HEX had the highest G-score (44), i.e. the highest propensity to form G4s. Because most of the pG4s in the VNTR region shared similar sequences, we selected three sequences, two in the antisense strand (VNTR1 and VNTR2) and one in the sense strand (VNTR3), for further analyses. For the HEX domain, we selected the minimal G4-forming motif (Hex), i.e. a sequence harbouring four repeats of the GGGAGA motif.

### *In vitro* characterisation of the XDP SVA G4s

To assess G4 folding topology and stability, the selected XDP SVA pG4 sequences were analysed by CD melting (Figure 1B and Supplementary Figure S1A). All the tested sequences displayed the CD signature of G4s with parallel topology, with a positive peak around 265 nm and high melting temperature (*T*_m_). In particular, the VNTR1 sequence showed exceptional stability (*T*_m_ > 90°C), while the Hex was the least stable G4, with *T*_m_ = 52.1°C (Supplementary Table S2). Overall, CD analysis showed that all tested sequences fold into stable G4s under physiological conditions. To identify the Gs involved in G4 formation, we performed DMS footprinting (Figure 1C and Supplementary Figure S1B). Oligonucleotides were folded in G4-destabilising (Li^+^) and G4-inducing (K^+^) conditions and treated with DMS to identify the Gs protected from DMS alkylation. In the unfolded conditions (lane U), all Gs were cleaved, as expected in an unstructured oligonucleotide. In the presence of K^+^ (lane F), protection at G4 residues was observed in all sequences, confirming G4 folding. In the Hex sequence, which presents the most regular G4 composition with four GGG-tracts, all G bases were involved in the formation of a three-quartet G4 structure (Figure 1D). The VNTR1 and VNTR3 sequences are composed of four G-tracts, while VNTR2 presents five G-tracts, all with different lengths ranging from two to seven Gs (Supplementary Figure S1C), which may lead to the formation of different G4 structures and conformations. Indeed, different degrees of protection were observed in all G-runs, including the shorter ones (Supplementary Figure S1C), suggesting dynamic folding in solution.

We next addressed the effect of the known G4 ligands (45), BRACO-19 (B19) (46) and quarfloxin (Q) (47) (Supplementary Figure S2), on the stability of G4s. The compounds mildly and highly increased the CD molar ellipticity at 25°C and *T*_m_, respectively, of the Hex G4 sequence (Figure 1E and Supplementary Table S2). CD molar ellipticity at 25°C was increased also with VNTR2 G4, while it decreased with VNTR1 and VNTR3; *T*_m_ of VNTR3 was highly increased by both G4 ligands, while *T*_m_ of VNTR2 was only mildly increased. VNTR1 was too stable even in the absence of G4 ligands to observe further stabilisation (Supplementary Figure S3A and Supplementary Table S2).

To evaluate the ability of the G4s to interfere with polymerase activity, we performed the *Taq* polymerase stop assay (*Taq* pol stop assay). We used the selected G4 sequences as templates (Supplementary Table S3) under different G4 stabilisation conditions, in the presence of K^+^ and G4 ligands. In general, K^+^ mildly affected polymerase progression, as indicated by a slight decrease in the full-length amplification product and the increase in the stop bands at the most 3′ G (Figure 1F and Supplementary Figure S3B). With both B19 and Q, polymerase blockage was significantly more pronounced at all G4 templates (Figure 1F and Supplementary Figure S3B).

Collectively, these results indicate that multiple G4s can form in the XDP SVA, and that all are greatly stabilised by G4 ligands.

### G4s formed in the genomic XDP SVA region

After having observed G4-dependent polymerase blockage on single-stranded DNA substrates *in vitro*, we set up a PCR stop assay (48) to test whether G4s can also form within the whole double-stranded SVA genomic region. We used gDNA extracted from hFib obtained from XDP patients (fXDP), from a non-manifesting carrier patient (fNMC), who inherited the XDP haplotype but had not yet developed the disease at the time of biopsy, and from healthy controls (fWT) (Table 1). In parallel, we also employed NPCs derived from patient (nXDP) and control (nWT) iPSC lines, as XDP is a neurological disease, and previous studies have shown that the transcriptional defects caused by the SVA insertion are most prominent in neural progenitors (8,29,30).

**Figure 2.**
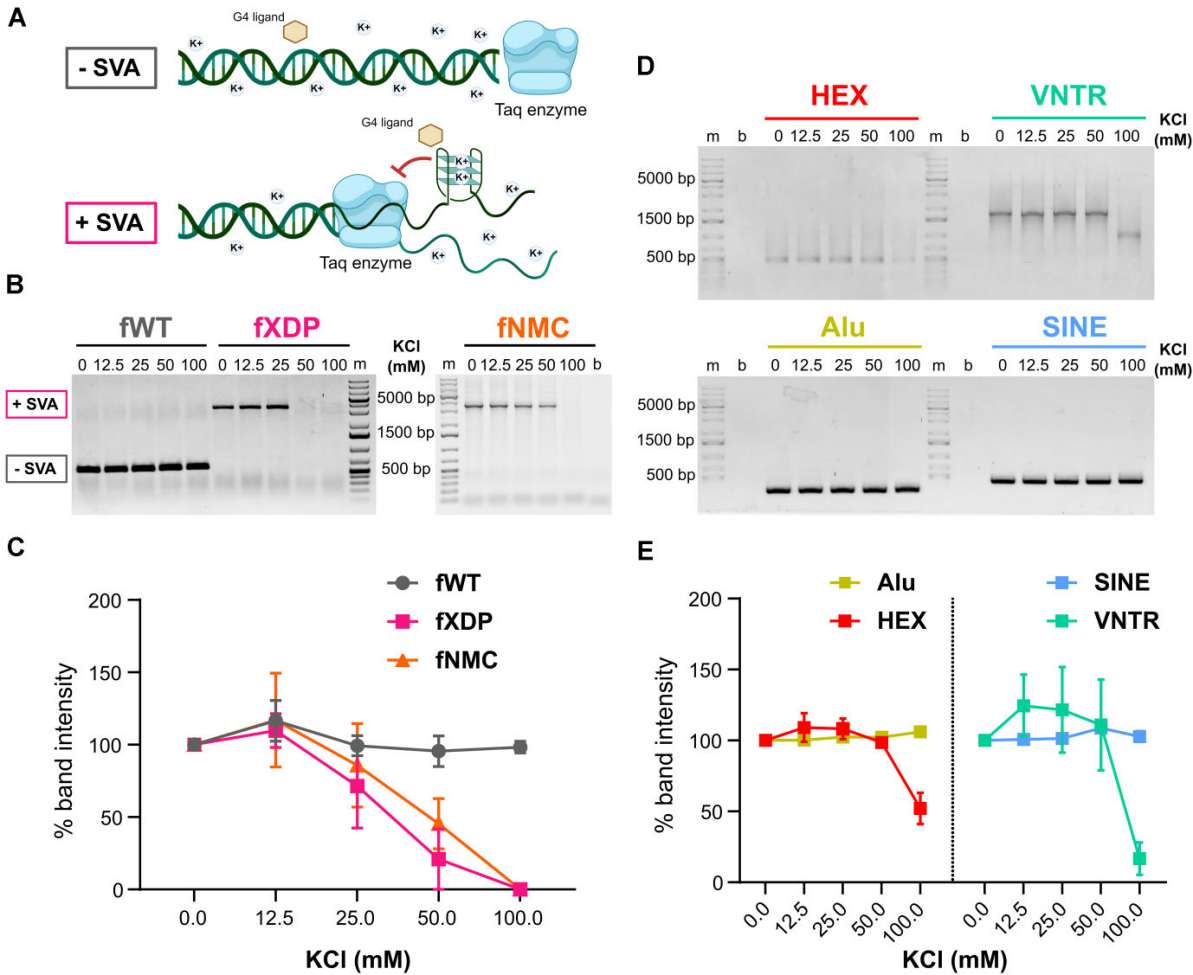
G4 stabilisation reduces polymerase activity at the VNTR and HEX domains of the XDP SVA. (**A**) Schematic illustration of the PCR stop assay. Under G4-inducing conditions, such as increasing KCl and G4 ligand concentrations, G4 folding of the G-rich sequences in the XDP SVA retrotransposon would block PCR amplification. The same conditions would not affect amplification of a non-G-rich template. (**B**) Representative SVA PCR stop assay agarose gel. Genomic DNA (gDNA) was extracted from XDP-affected, NMC and healthy wild-type (WT) cells, folded at different KCl concentrations, and used as PCR template. (**C**) Quantification of the gel bands shown in panel (B). Intensity of the bands from the PCR reactions was quantified with ImageJ and normalised on the sample without KCl. G4 stabilisation by increasing KCl concentration correlates with a decrease in PCR product in gDNA containing the SVA insertion, but not in controls. Error bars represent standard deviation (*n* = 2). (**D**) Representative agarose gel of the nested PCR stop assay for each SVA domain at increasing concentrations of KCl. Only the two putative domains where pG4s were predicted, i.e. VNTR and HEX, show less amplification product at 100 mM KCl. Amplification of the Alu and SINE domains is not reduced. (**E**) Quantification of the gel bands shown in panel (D). Only HEX and VNTR amplification is impaired at increasing KCl concentrations, confirming the presence of G4 structure within the two domains. Error bars represent standard deviation (*n* = 2).

We performed PCR using primers flanking the SVA insertion, according to the approach that has been previously used to amplify all the XDP SVA variants and determine the XDP genotype (31) (Figure 2A). We obtained an amplification band at about 600 bp in the control DNA and an upward shifted band at about 3200 bp in XDP and NMC gDNA (600 + 2600 SVA bp), consistent with SVA insertion (Supplementary Figure S4). We next used G4 stabilising conditions (i.e. K^+^ and G4 ligands) to assess whether G4s in the double-stranded SVA would impair polymerase processivity and thus reduce the amount of the PCR amplicon. With increasing concentrations of K^+^ (Figure 2B and C) and G4 ligands (Supplementary Figure S5), we observed strong and concentration-dependent reduction of the intensity of the band corresponding to the SVA full-length amplicon from XDP cells. In contrast, the amplification from control samples was not affected, as expected since no G4-forming sequences were predicted by QGRS in the amplified region (Supplementary Figure S6).

To determine the SVA domains in which amplification was blocked, we set up a nested PCR stop assay (Figure 2D and E). We designed domain-specific primers to amplify each individual SVA domain using the purified SVA amplicon as a template (Supplementary Table S4 and Supplementary Figure S7). We found that the two most G4-rich domains, HEX (red) and VNTR (green), were the only ones where PCR amplification was blocked under G4-inducing conditions, in line with our bioinformatic and *in vitro* analyses. Accordingly, amplification of the SINE (blue) and Alu domains (mustard) that were predicted to form zero and one G4, respectively (Supplementary Table S1), was not affected.

Overall, the analysis of the genomic XDP SVA locus confirmed that G4s readily form in the HEX and VNTR domains and that they are sufficiently stable to interfere with polymerase progression *in vitro*.

### G4s formed in SVAs in cells

**Figure 3.**
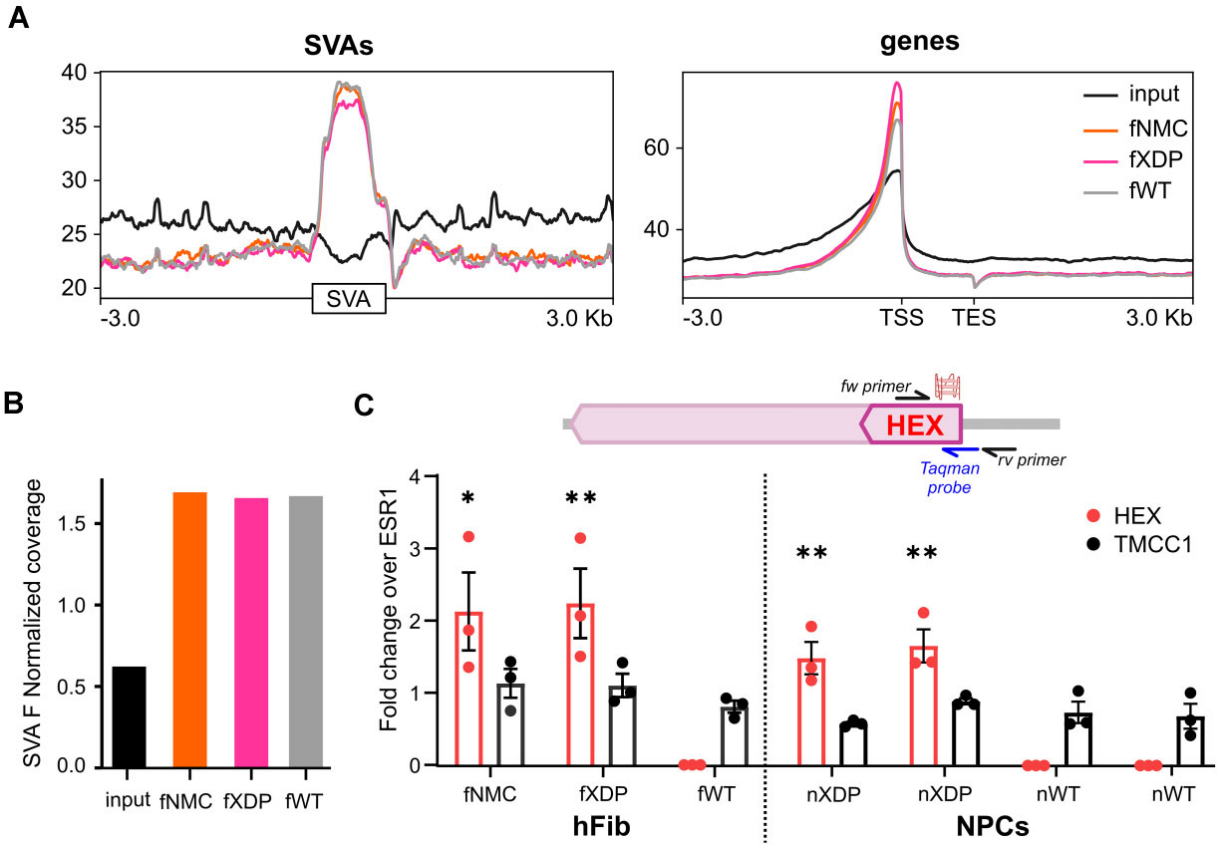
G4s form in the XDP SVA in XDP hFib and NPCs. (**A**) Plot profiles of G4 ChIP-seq from hFib. Representative G4-ChIP-seq plot profiles showing G4 enrichment in SVA retrotransposons compared with the input signal (left panel). G4-ChIP-seq plot profiles at gene TSS (right panel) corroborate literature data in other cell lines (50). Input profile is shown in black, fNMC in orange, fXDP in pink and fWT in grey. All three G4-ChIP profiles are enriched compared with the input. (**B**) G4-ChIP-seq coverage in the SVA F retrotransposon family. The bar plot represents the read coverage obtained in the input, fNMC, fXDP and fWT profiles. The increase in G4-ChIP-seq coverage confirms that SVA F retrotransposons, such as the XDP SVA, contain G4s that are folded in cells. (**C**) G4-ChIP-qPCR at the XDP SVA HEX region in hFib and NPCs. Results are reported as enrichment over the G4-negative region ESR1. The XDP SVA HEX region is shown in red and the G4-negative region TMCC1 in black. Only SVA carrier cells show enrichment of the HEX, which is higher compared with the negative ctrl TMCC1, confirming the presence of G4s in XDP cells within the XDP SVA HEX. Significance levels were calculated using ANOVA test (*n* = 3) considering ctrl hFib or NPCs as reference for HEX and TMCC1 genes (ns = *P* ≥ 0.05, * *P* < 0.05, ** *P* < 0.01).

Our *in vitro* data indicate that several highly stable G4s can form within the XDP SVA. We therefore set out to evaluate G4 folding at this locus in cells by G4 ChIP-seq (17). The BG4 antibody was specifically designed to detect G4s (16), and G4-ChIP with the BG4 antibody is a well-established technique for genome-wide mapping of G4 structures in cells (19,33), where G4s have been found highly enriched at transcription start sites (TSSs) (17). We compared the G4-ChIP-seq coverage on SVAs and TSSs in fXDP using deepTools (37). Both SVAs and TSSs were enriched in the G4-ChIP-seq profile, with a better coverage at the TSS, probably due to the higher mappability of these genomic regions compared with repetitive elements, like SVAs (Figure 3A). These data suggest that SVAs overall harbour G4s in cells. However, due to the highly repetitive nature of this class of retrotransposons, it is difficult to obtain unambiguous mapping to the single entity (49). We thus first performed family enrichment analysis with Homer suite (38) and observed enrichment of the SVA F family, which XDP SVA belongs to, in all G4 ChIP-seq samples compared with the input (Figure 3B).

Encouraged by these results, we performed G4-ChIP-qPCR with primers specific for the HEX domain of the XDP SVA to assess G4 formation exclusively at the XDP SVA. We designed one primer complementary to the HEX repeat and one to the flanking region downstream of the XDP SVA insertion. Consistently with the specificity to the XDP SVA, this design yielded amplification only in XDP cells. To quantify G4 enrichment, we calculated the fold change over ESR1, a reported G4-negative region (17,33), of both HEX and TMCC1, the latter being another well-established G4-negative region (19) common to both XDP and control cell lines. We observed significantly higher G4 enrichment at HEX compared with TMCC1 in both hFib and NPCs, in all tested samples, including the NMC fibroblasts (Figure 3C). These data indicate that G4s are present in their folded state in the HEX domain of the XDP SVA in the genome of XDP-affected cells.

**Figure 4.**
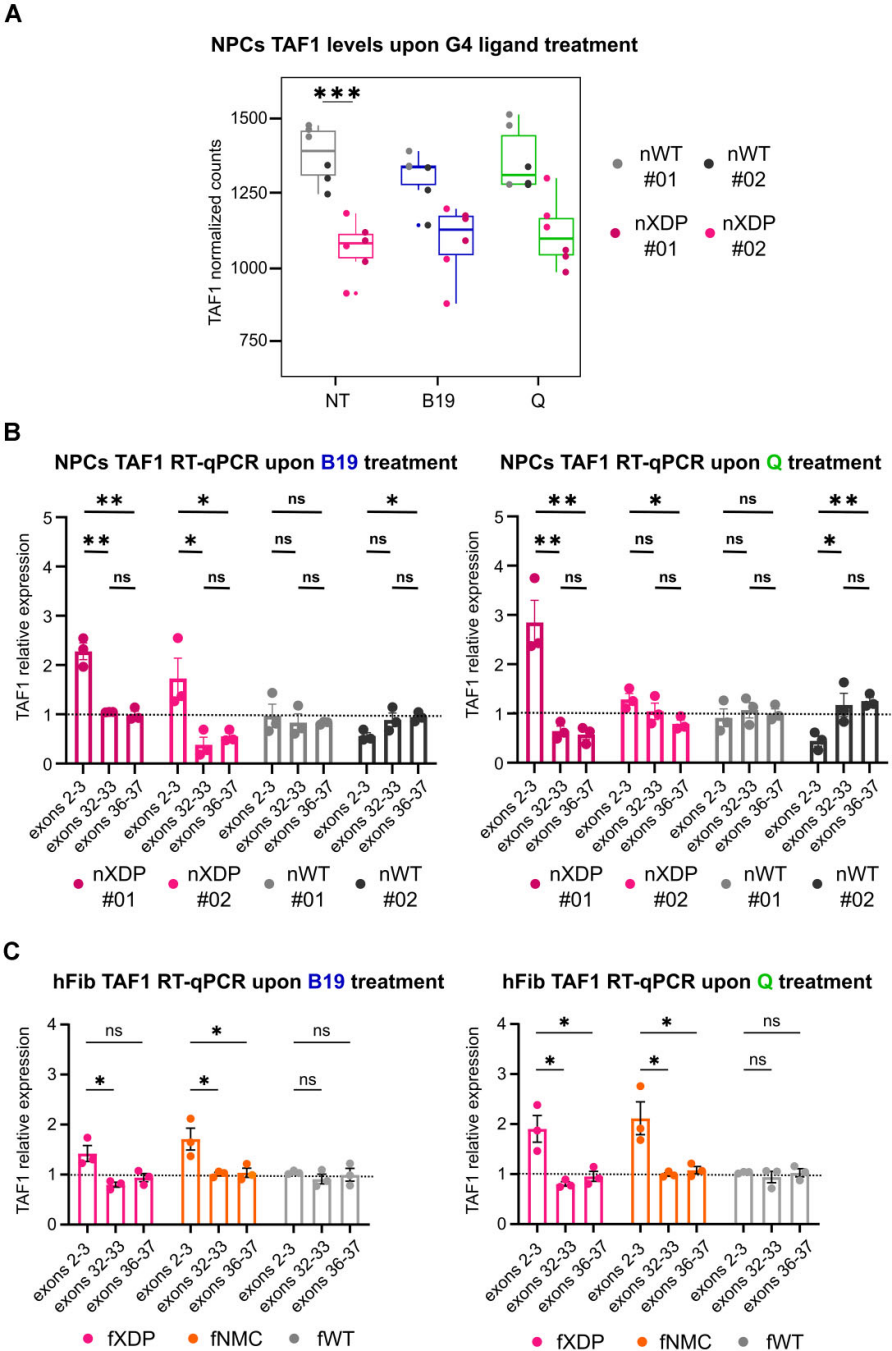
*TAF1* transcript levels in XDP cells. (**A**) *TAF1* transcript levels in NPCs untreated (NT) or treated with G4 ligands B19 and Q from RNA-seq analysis. Statistical analysis was performed with the non-parametric simple *t*-test (*n* = 3). Transcript levels of different exons of *TAF1* upon treatment with G4 ligands (B19 5 μM or Q 3 μM) in NPCs (**B**) and hFib (**C**) by RT-qPCR analysis. The transcript levels of each exon were normalized to the housekeeping gene β-actin and to the untreated sample (*n* = 3). G4 ligand treatment decreased *TAF1* transcription of the exons flanking and downstream of the SVA and increased transcription of the most upstream exons in XDP cells compared with WT cells.

### G4 stabilisation affects TAF1 transcripts in XDP cells

One of the main features characterising XDP cells is the lower *TAF1* expression compared with WT cells (Figure 4A and Supplementary Figure S10). In particular, expression of *TAF1*’s most 3′ exons is strongly downregulated, mainly in neuron-like cells (8). We reasoned that this phenotype is compatible with the folded XDP SVA G4s acting as roadblocks during transcription and triggering RNA polymerase II stalling at the SVA within the *TAF1* gene.

To assess the effect of XDP SVA G4s on *TAF1* transcription, we performed RNA-seq and RT-qPCR in cells treated with G4 ligands. We hypothesised that stabilisation of G4s in the SVA by the ligands would lead to a decreased expression of *TAF1* mRNA. We first assessed cytotoxicity of the two G4 ligands B19 and Q to establish a range of sub-cytotoxic concentrations in both XDP hFib and NPCs (Supplementary Figure S9). For each compound, we used the highest non-toxic concentration in both cell types (5 μM for B19, 3 μM for Q) and we recovered RNA after 24 h treatment. We expected to observe a considerable decrease in *TAF1* expression in XDP cells compared with WT cells after treatment with G4 ligands but did not observe any consistent decrease upon B19 and Q treatment (Figure 4A and Supplementary Figure S10), while we obtained a mild G4 ligand-dependent decrease in full-length taf1 in both NPCs and hFib control cells. We attributed this effect to XDP-unrelated G4-mediated pathways triggered by B19 (51), resulting in a global decrease in *TAF1* transcription.

Given this unexpected outcome, we reasoned that quantifying full-length *TAF1* transcripts upon treatment with G4 ligands might underestimate the effect of ligands on *TAF1* transcription for two reasons. First, Taf1 is a cofactor of RNA polymerase II and the level of activation of *TAF1* transcription is highly controlled by the cell. Second, G4-mediated inhibition on *TAF1* levels in XDP cells could lead to the production of *TAF1* transcripts truncated near the SVA insertion, which would be recognised as aberrant mRNA and thus degraded by the RNA degradation machinery (52).

We thus refined our analysis to perform RT-qPCR on single exons of the *TAF1* transcript, using the random hexamers for the RT reaction, so to detect also the intermediate *TAF1* transcript isoforms. As XDP SVA is inserted in intron 32 of *TAF1*, we evaluated the expression levels of *TAF1* exons upstream (exons 2 and 3), around (exons 32 and 33) and downstream (exons 36 and 37) of the SVA insertion by RT-qPCR. We observed a double effect: transcription of the exons downstream and at the SVA insertion was downregulated in both XDP NPCs and XDP hFib upon G4 ligand treatment (Figure 4B and C). At the same time, we observed a significant increase in the transcription of the first exons of *TAF1* in XDP cells (Figure 4B and C). These effects were slightly more pronounced in NPCs than in hFib. We further confirmed this observation by measuring the levels of individual exons in NPCs by RNA-seq analysis (Supplementary Figure S11A and B). Again, the two G4 ligands showed a similar behaviour in XDP NPCs, where they increased transcription of exons upstream of the SVA site only in XDP cells. In contrast, no difference or decrease was observed in WT cells (Supplementary Figure S11A and B). These observations are consistent with a modulatory effect of G4 ligands on *TAF1* transcription, through binding to the G4s formed at the SVA.

**Figure 5.**
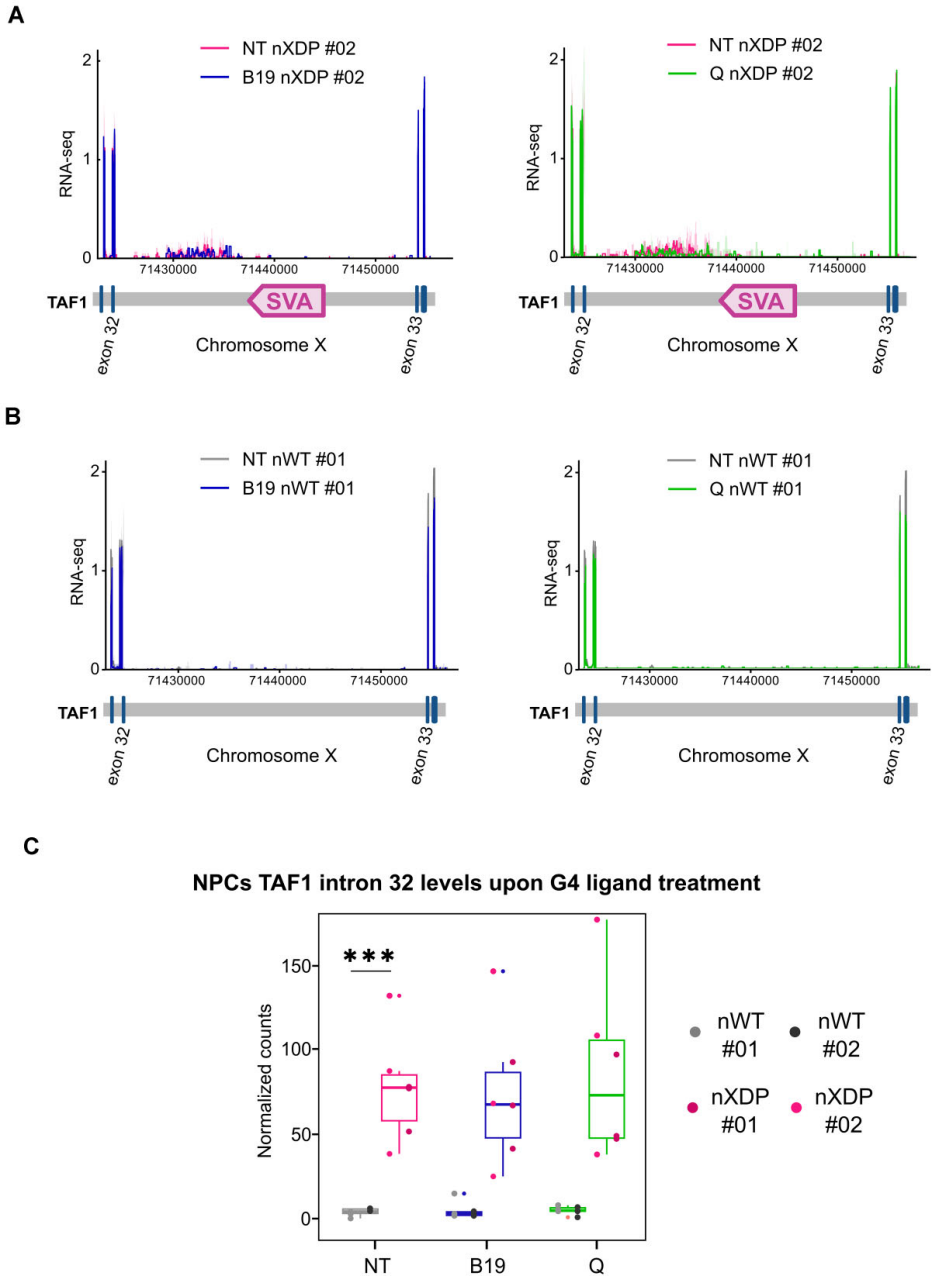
*TAF1*intron 32 retention levels in XDP and WT NPCs. (**A**) *TAF1* RNA-seq profile of G4 ligand treated XDP cell line with B19 (left panel) or Q (right panel). Untreated (NT) nXDP NPC cell line (pink). RNA-seq profile presented is the mean profile of *n* = 3 biological experiments. (**B**) *TAF1* RNA-seq profile of G4 ligand treated nWT cell line with B19 (left panel) or Q (right panel). Non-treated (NT) nWT NPC cell line (grey). RNA-seq profile presented is the mean profile of *n* = 3 biological experiments. (**C**) RNA-seq *TAF1* intron 32 coverage in NPCs upon G4 ligand treatment. Intron 32 coverage is present only in nXDP cells. Statistical analysis was performed with the non-parametric simple *t*-test (*n* = 3).

We next asked whether G4 ligand treatment would induce alternative splicing and intron retention. We analysed our RNA-seq data obtained in both NPCs and hFib and found that, while a few genes were alternatively spliced upon G4 ligand treatment in both cell lines, no such events were present in *TAF1* transcripts (Supplementary Figures S12 and S13). We observed mild intron 32 retention only in the XDP cell lines, as previously reported (8): this involved only the intron region just upstream of the SVA insertion (Figure 5A–C and Supplementary Figures S14 and S15). Upon treatment with G4 ligands, there was no significant variation in the intron 32 retention signal (Figure 5C). These data suggest that intron retention or other alternative splicing events are not the cause of the increased transcription of the *TAF1* exons upstream of the SVA site.

### G4 destabilisation rescues TAF1 downregulation

**Figure 6.**
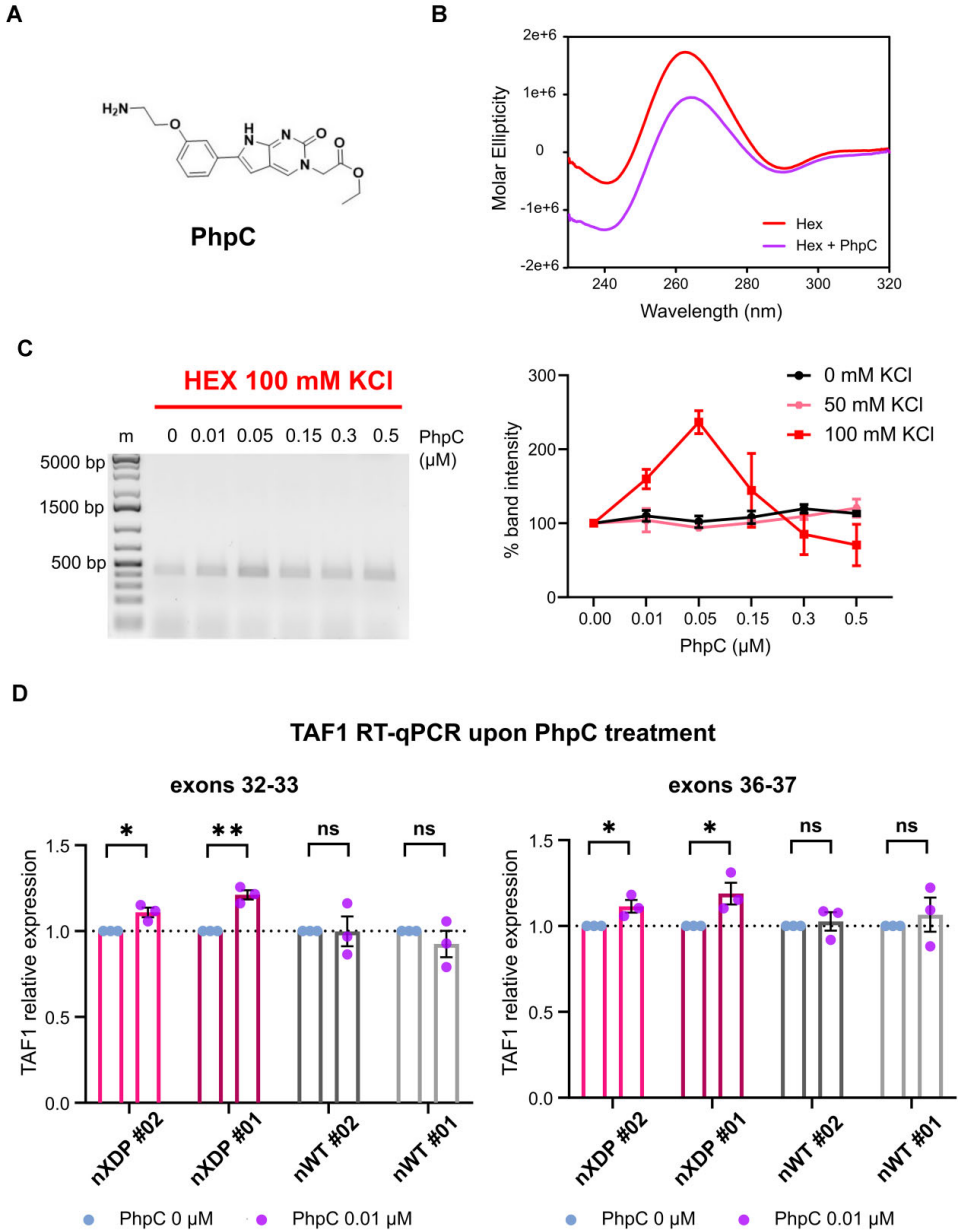
PhpC destabilises G4 Hex. (**A**) Molecular structure of PhpC. (**B**) CD analysis of Hex G4 alone (red) or with the G4 destabiliser PhpC (purple). CD spectra were recorded at 25°C: they show a parallel G4 topology with a positive peak at 265 nm. PhpC spectra show a decrease in molar ellipticity, indicating destabilisation of the G4 structure. (**C**) Representative agarose gel of a nested PCR stop assay for the HEX domain in 100 mM KCl (left panel). Amplification increases at lower compound concentrations. Quantification of the gel bands of the nested PCR stop assay at different KCl concentrations (right panel). Only at 100 mM KCl PhpC shows an increase in amplification at low concentration. No such effect is observed at 0–50 mM KCl. Error bars represent the standard deviation (*n* = 2). (**D**) RT-qPCR of exons flanking and following SVA insertion in XDP and WT NPCs treated for 24 h with 0.01 μM PhpC (*n* = 3). A small but significant increase in transcription of these exons is observed, but only in nXDP cell lines. nWT cells were unaffected. Statistical analysis was performed using the non-parametric sample *t*-test (ns = *P* ≥ 0.05, *P < 0.05, **P < 0.01). Error bars represent the standard deviation. Results in XDP cells are shown in pink and control cells in grey. Treated samples are depicted in purple and non-treated samples in light blue.

If G4s are responsible for blocking RNA polymerase transcription of the *TAF1* gene, then G4 unfolding would help restore transcription. We thus took advantage of PhpC (53–55), one of the very few small molecule available to unfold G4s *in vitro* and in cells (Figure 6A), to destabilise the G4s within the XDP SVA. First, we used CD to determine whether PhpC was able to destabilise the Hex G4 (Figure 6B). Molar ellipticity decreased in the presence of the compound, indicating that fewer G4s were folded. We thus tested the unfolding ability of PhpC on the entire HEX domain by performing a nested PCR stop assay (Figure 6C and Supplementary Figure S16). We observed increased band amplification, indicating G4 destabilisation, only at 100 mM KCl, i.e. the condition where HEX G4s are folded. The highest destabilising activity was achieved only at a specific range of low concentrations, suggesting a tight concentration-dependent mechanism of action. These results indicate that low concentrations of PhpC can destabilise the G4s within the HEX domain, thereby facilitating polymerase processing, in line with previously reported *in vitro* results. We therefore tested PhpC on XDP and WT NPCs at 0.01 μM, PhpC being non-cytotoxic in these cell lines up to 200 μM (Supplementary Figure S17): we measured the levels of exons flanking and downstream of the SVA insertion after 24 h of treatment by real-time qPCR and observed a small but significant increase in *TAF1* transcription in the XDP cell lines and not in WT cells (Figure 6D). These results indicate that unfolding of the G4s within the XDP SVA helps rescue the XDP phenotype by restoring *TAF1* levels.

## Discussion

We have shown here that G4s form in the XDP SVA, the genetic variant specific to XDP. It has previously been shown that insertion of the SVA into intron 32 of the *TAF1* gene reduces the levels of full-length *TAF1* transcripts, whereas deletion of the SVA in cells derived from XDP patients restores normal *TAF1* levels (8), but the underlying causes remain unclear. Here, we provide the first evidence that XDP SVA G4s do fold in cells and that their stabilisation disrupts *TAF1* transcription, leading to both decrease in full-length *TAF1* transcripts and increase in short transcripts (Figure 4B and C). These events did not depend on alternative splicing events or intron retention, which are not present in the *TAF1* gene even upon treatment with G4 ligands (Figure 5 and Supplementary Figures S12–S15). The inhibitory effect of the SVA G4s on transcription is consistent with the presence of G4s, such as those identified in the HEX domain. Stable G4s would indeed be prone to temporarily stall RNA polymerase, especially if present in large numbers, like the G4s in the whole HEX domain, which are largely increased due to Hex repeat expansions. In support of this model, the number of repeat expansions in XDP patients has been shown to adversely affect XDP disease severity and age of onset (8,10).

The involvement of the SVA G4s is supported by the fact that *TAF1* transcripts were downregulated at and downstream of the SVA insertion, which is in perfect agreement with the block of polymerase progression observed at the G4s *in vitro* (Figure 2). In contrast, the first exons of the *TAF1* transcript were upregulated: we ascribe this effect to a positive feedback mechanism, whereby a decrease in full-length *TAF1* transcripts caused by RNA polymerase stalling at the G4s in the SVA is perceived by the cell as a signal to increase *TAF1* transcription, leading to the observed accumulation of short, incomplete *TAF1* transcripts in XDP cells (8). This effect was more pronounced in NPCs than in fibroblasts (compare Figure 4B and C). Since XDP NPCs have lower steady-state levels of *TAF1* transcripts (Figure 4A), we reasoned that an additional reduction induced by the G4 ligand treatment would intensify the positive feedback mechanism in NPCs, thereby amplifying the difference in transcript levels between exons upstream and downstream of the XDP SVA.

To study the effect of G4 stabilisation, we used two well-known G4 ligands, each with a unique core: the acridine B19 (56) and the fluoquinolone Q (47). Q was more efficient than B19 in stabilising the G4s formed in the single-stranded oligonucleotides (Supplementary Table S2). However, when the G4s were embedded in a more extended oligonucleotide environment (i.e. *Taq* pol stop assay, Figure 1F and Supplementary Figure S3), or in a double-stranded DNA context (i.e. the PCR stop assay, Supplementary Figures S5 and S8), Q and B19 showed similar activity. Since all SVA G4s are folded in the parallel conformation, implying similar interaction with the G4 ligands, it is possible that the enhanced stabilisation observed for Q at the single strand level is due to Q interaction at the free oligonucleotide ends, as reported for the c-MYC promoter G4 (57). In XDP cells, the two G4 ligands showed similar cytotoxicity and activity (Supplementary Figure S8). Since multimeric G4s can form in the HEX region, as reported for the telomeric and some promoter sequences (58), it is possible that the interaction of the G4 ligands with the multimeric HEX G4s is favoured, even though B19 interaction with the multimeric telomeric G4s was reported to be low (59).

There are only a very few options to unfold G4s, which can be achieved either *in situ* expressing G4 helicases or using small molecule unfolders (60). We used one of the very few available chemicals, PhpC (53,61), found here to exert an unfolding activity in all our tests, *in vitro* and in cells. Interestingly, the range of concentrations is key to obtain the unfolding activity, as high PhpC may preclude access to G4 clusters instead of ironing them out, thus hindering proper enzyme activity. Admittedly, and in line with previous results, the effect of PhpC in our systems was weak both *in vitro* (CD analysis, where only a fraction of the HEX G4s was unfolded by PhpC) and in cells (e.g. the PhpC-mediated increase in *TAF1* transcription). This could be attributed to both the weak activity of PhpC *per se* and the modest fraction of transcripts that are normally paused/stopped in our cell growing conditions, as shown by the levels of *TAF1* transcripts in WT and XDP cells (Figure 4A and B). Hence, PhpC exerts its destabilising effect on this fraction of transcripts only, explaining why the overall effect is expected to be moderate. We anticipate that *in vivoTAF1* short transcript accumulation and decreased Taf1 protein levels would damage and not sustain basic pathways in neurons over time: in this context, preventive treatment with PhpC, which has been shown to be non-cytotoxic in human astrocytes (55), may prevent the development of XDP symptoms.

The main elements of our study converge towards genetic instability, G4s on one side, already implicated in genome instability (62,63), and the XDP SVA on the other side, prone to somatic instability (64,65) probably as a result of mutations in DNA repair genes that have been identified in the region of the SVA insertion (66). This is further substantiated by the somatic variability in the number of HEX repeats within the XDP SVA, which show heterogeneity even within different brain regions of the same patient (67) and may also suggest the involvement of DNA repair-mediated mechanisms. Given that *TAF1* is a highly transcribed gene and that most of the affected neurons show somatic instability that is independent of DNA replication, we propose here that transcription-coupled DNA repair may be involved in XDP SVA. We therefore speculate that the G4s present in the XDP SVA within the *TAF1* gene may be the main cause not only of the reduced *TAF1* expression, but also of the genomic instability leading to the genetic HEX repeat expansion observed in XDP, as in other repeat expansion-related diseases (68). In addition, Taf1 has been shown to interact directly with p53 in cell cycle regulation, and thus the alterations in *TAF1* levels observed in XDP patients may contribute to the defects in DNA repair (69).

Our ChIP-seq data suggest that G4s may also be present in other SVAs within the human genome. Although it is not possible to determine their location with the present analysis, it is noteworthy that very few pathologies have been reported to be associated with SVAs (70–74). One possible explanation is that most genome-wide SVAs are epigenetically silenced by DNA methylation and repressive histone marks (75), in addition to the many specific SVA-silencing protein complexes, such as the Krüppel-associated box domain-containing zinc finger proteins (76,77). DNA methylation at the XDP SVA has recently been reported to enhance *TAF1* expression (78). As methylation reduces G4 formation (79), these findings are complementary and in line with those reported here. Conversely, the few disease-associated SVAs have always been reported within actively transcribed genes (44), where their altered regulation or alternative splicing might be mediated by SVA G4s. In such a context, retrotransposon silencing may be hindered, thus allowing G4s to form more easily.

In the case of XDP, we propose that the insertion of an SVA into the *TAF1* gene creates defects in a multipronged, multisectoral and multidimensional way: the high transcription rates required to maintain Taf1 production collide with G4 formation, which on the one hand slows down the process and on the other hand increases recombination and genome instability (80). The latter events are mainly dependent on R-loop formation when the G4s are in the sense (non-template) strand), as in the case of the HEX G4s, but have also been reported to occur, albeit to a lesser extent, even when the G4s are formed in the antisense (template) strand, as in the case of the VNTR G4s (81,82). If unrepaired, the accumulation of DNA damage leads to cell death. In such a scenario, the limited ability of neurons to proliferate would exacerbate the problem, leading to atrophy of brain areas, as is indeed observed in XDP (3).

Finally, our results also open an interesting way to alleviate XDP phenotype, as we show that small molecules able to unfold the XDP SVA G4s, PhpC here but potentially others candidates as well (53,83,84), may prove successful in treating the disease *in vivo*. Research in this area is therefore urgently needed to provide tangible help to XDP patients.

## Supplementary Material

gkae797_Supplemental_File

## Data Availability

Sequencing raw data and processed data have been deposited in the NCBI GEO database under the accession number GSE250295.
