# Supplementary material for: G-quadruplexes in an SVA retrotransposon cause aberrant TAF1 gene expression in X-linked dystonia parkinsonism
Source: Nucleic Acids Res. 2024 Sep 17;52(19):11571–86. doi: 10.1093/nar/gkae797 (PMC12053379; doi:10.1093/nar/gkae797)
Supplement: gkae797_Supplemental_File [file gkae797_supplemental_file.pdf]

Supplementary Information

G-quadruplexes in an SVA retrotransposon cause aberrant *TAF1* gene expression in X-linked Dystonia Parkinsonism

Giulia Nicoletto,[1] Marianna Terreri,[1†] Ilaria Maurizio,[1†] Emanuela Ruggiero,[1] Filippo M. Cernilogar,[2,3] Christine A. Vaine,[4] Maria Vittoria Cottini,[1] Irina Shcherbakova,[3] Ellen B. Penney,[4] Irene Gallina,[1] David Monchaud,[5] D. Cristopher Bragg,[4] Gunnar Schotta,[3] Sara N. Richter[1,6*]

[1]Department of Molecular Medicine, University of Padua, 35121 Padua, Italy
[2]Department of Science and Technological Innovation, University of Piemonte Orientale, Alessandria, Italy
[3]Molecular Biology Division, Biomedical Center, Ludwig-Maximilians-University Munich, Planegg-Martinsried 82152, Germany
[4]Department of Neurology, Massachusetts General Hospital, Charlestown, MA 02129 USA
[5]Institut de Chimie Moleculaire, Universite de Dijon, ICMUB CNRS UMR6302, 21078, Dijon, France
[6]Microbiology and Virology Unit, Padua University Hospital, 35121 Padua, Italy

Table S1: **Predicted putative G4 (pG4) sequences within the SVA retrotransposon using the QGRS prediction tool.** For every pG4 sequence found, the SVA domain, exact position, length, sequence, G-score and name are given. Gs that may be involved in the G4 structure are highlighted in bold.

SVA domain		Position	Length	Sequence	G - Score	Name
TAF1 sense strand	Poly T	-	-	-	-	-
	SINE	-	-	-	-	-
	VNTR	617	23	**GGG**C**GG**CT**GG**CC**GGG**C**GGGGGGG**	64	VNTR1
		747	23	**GGG**CCACT**GG**CC**GGG**CA**GGGGGG**	63	VNTR2
		798	22	**GGG**C**GG**CT**GG**CC**GGG**C**GGGGGG**	63	
		1346	23	**GGG**C**GG**CT**GG**CC**GGG**T**GGGGGGG**	64	
		1572	22	**GGG**C**GG**CT**GG**CC**GGG**T**GGGGGG**	63	
	Alu	2466	26	**GGG**AGACCGTG**GGG**AGA**GGG**AGA**GGG**	68	
	HEX	2498	21	**GGG**AGA**GGG**AGA**GGG**AGA**GGG**	72	HEX
TAF1 antisense strand	HEX	-	-	-	-	-
	Alu	-	-	-	-	-
	VNTR	1015	20	**GGG**A**GGG**A**GG**T**GGGGGGGGG**	69	
		1066	17	**GGG**A**GGG**A**GG**T**GGGGGG**	68	VNTR3
		1115	18	**GGG**A**GGG**A**GG**T**GGGGGGG**	69	
		1242	18	**GGG**A**GGG**AGAT**GGGGGGG**	69	
	SINE	-	-	-	-	-
	Poly A	-	-	-	-	-

Table S2: Melting temperature (T_m) of SVA G4s when folded into G4 and in the presence of the G4 ligands BRACO-19 (B19) or quarfloxin (Q). For each tested sequence T_m is reported in KCl 10 or 100 mM.

oligo	T_m KCl (°C)	T_m B19 (°C)	T_m Q (°C)	ΔT_m B19 (°C)	ΔT_m Q(°C)
HEX	52.1 ± 0.5	68.2 ± 1.5	>90	16.1	>37.9
VNTR1	>90	>90	>90	ND	ND
VNTR2	65.9 ± 2.3	74.6 ± 3.9	77.8 ± 2.1	8.7	11.9
VNTR3	54.9 ± 1.4	>90	>90	> 35.1	> 35.1

Table S3: Oligonucleotide sequences used in DMS-footprinting and TaqPol stop assays.

oligo	Sequence (5'-3')
HEX footprinting	TTTTTAGAGGGAGAGGGAGAGGGAGAGGGTTTTT
VNTR1 footprinting	TTTTTACGGGGCCACTGGCCGGGCAGGGGGGCTTTTTT
VNTR2 footprinting	TTTTTACAGGGCGGCTGGCCGGGCGGGGGGCTTTTTT
VNTR3 footprinting	TTTTTTCCGGGAGGGAGGTGGGGGGGTCTTTTT
HEX Taq	TTTTTAGAGGGAGAGGGAGAGGGAGAGGGCTTTTTCTGCATATAAGCAGCTGCTTTTTGCC
VNTR1Taq	TTTTTACGGGGCCACTGGCCGGGCAGGGGGGCTTTTTTCTGCATATAAGCAGCTGCTTTTTGCC
VNTR2 Taq	TTTTTACAGGGCGGCTGGCCGGGCGGGGGGCTTTTTTCTGCATATAAGCAGCTGCTTTTTGCC
VNTR3 Taq	TTTTTTCCGGGAGGGAGGTGGGGGGGTCTTTTTCTGCATATAAGCAGCTGCTTTTTGCC

Table S4: Nested PCR amplification protocol and primer sequence for each XDP SVA domain.

name	Sequence (5-3')	PCR protocol
SVA - 16153 F	GTTCCATTGTGTGGTTGTACCAGCGTTTGTTC	94 °C 2min, 30x (98 °C 10s, 68 °C 3min 30s), hold at 8°C
SVA -19345 R	CACATGAAAAGATGCCCAACATCATTAGCCATTAG	
Hex for	AGCAGTACAGTCCAGCTTTGGC	94 °C 2min, 30x (98 °C 10s, 68 °C 20s), hold at 8°C
Hex rev	CTCAAGCCTTATTACAATGCCAGT	
VNTR 1 rev	AATCTTTTCCCCGCCTTTCC	94 °C 2min, 30x (98 °C 10s, 68 °C 1min 45s), hold at 8°C
VNTR 1 for	TCACTACAACCCACACCTCC	
SINE 1 for	ATAGTGGAGGGAAGGTCAGC	94 °C 2min, 30x (98 °C 10s, 60 °C 15s, 68 °C 30s), hold at 8°C
SINE 1 rev	GTGCCCAACAGCTCATTGAG	
Alu 1 for	GGCACCATTGAGCACTGAG	94 °C 2min, 30x (98 °C 10s, 55°C 15s, 68 °C 25s), hold at 8°C
Alu 1 rev	CCACGGTCTCCCTCTCATG	

Table S5: List of primers for G4-ChIP-qPCR

primer	Sequence (5'-3')
G4_ChIP_Hex_fw	GAGGGAGAGGGAGAG
G4_ChIP_Hex_rv	CGTTCATGTGTGAGATG
G4_ChIP_Hex_pr	FAM-CCTCAAGCCTTATTACAATGCCAGT -TAMRA
G4_ChIP_ESR1_fw	GAAACAGCCCCAAATCTCAA
G4_ChIP_ESR1_rv	TTGTAGCCAGCAAGCAAATG
G4_ChIP_ESR1_pr	FAM - AGTGGCACCCAGACTTGATGGCCGAC - TAMRA
G4_ChIP_TMCC1_fw	GTGGTACACTGCCTACAGTATT
G4_ChIP_TMCC1_rv	GTATAACGCCTGGGCTATGT
G4_ChIP_TMCC1_pr	FAM - TGGCTGCTCCTCCCTTGTGCTG -TAMRA

Table S6: List of primers for TAF1 RT-qPCR

primer	Sequence (5'-3')
TAF1_ex2-3_fw	AAGAATTGACCGGGACTGAC
TAF1_ex2-3_rev	CTTCGGCTTTCATCTTCTGC
TAF1_ex2-3_probe	FAM-ACCCACCCTTCATCATTT-TAMRA
TAF1_ex32-33_fw	ACCTTATTCTGGCCAACAGTGTT
TAF1_ex32-33_rev	ACAATCTCCTGGGCAGTCTTAGTAT
TAF1_ex32-33_probe	FAM-ACTCTCAGGTCCATTATAC-TAMRA
TAF1_ex36-37_fw	GGAGTGATGAAGAAGGAG
TAF1_ex36-37_rev	GGTTGTTTGGGTGTTATTC
TAF1_ex36-37_probe	FAM-CCACATCAGAGTCACTTCCACT-TAMRA
b-Actin_fw	TCACCGAGCGCGGCTACA
b-Actin_rev	CCTTAATGTCACGCACGATTTC
b-Actin_probe	FAM-TCACCACCACGGCCGAGCG-TAMRA

Figure S1

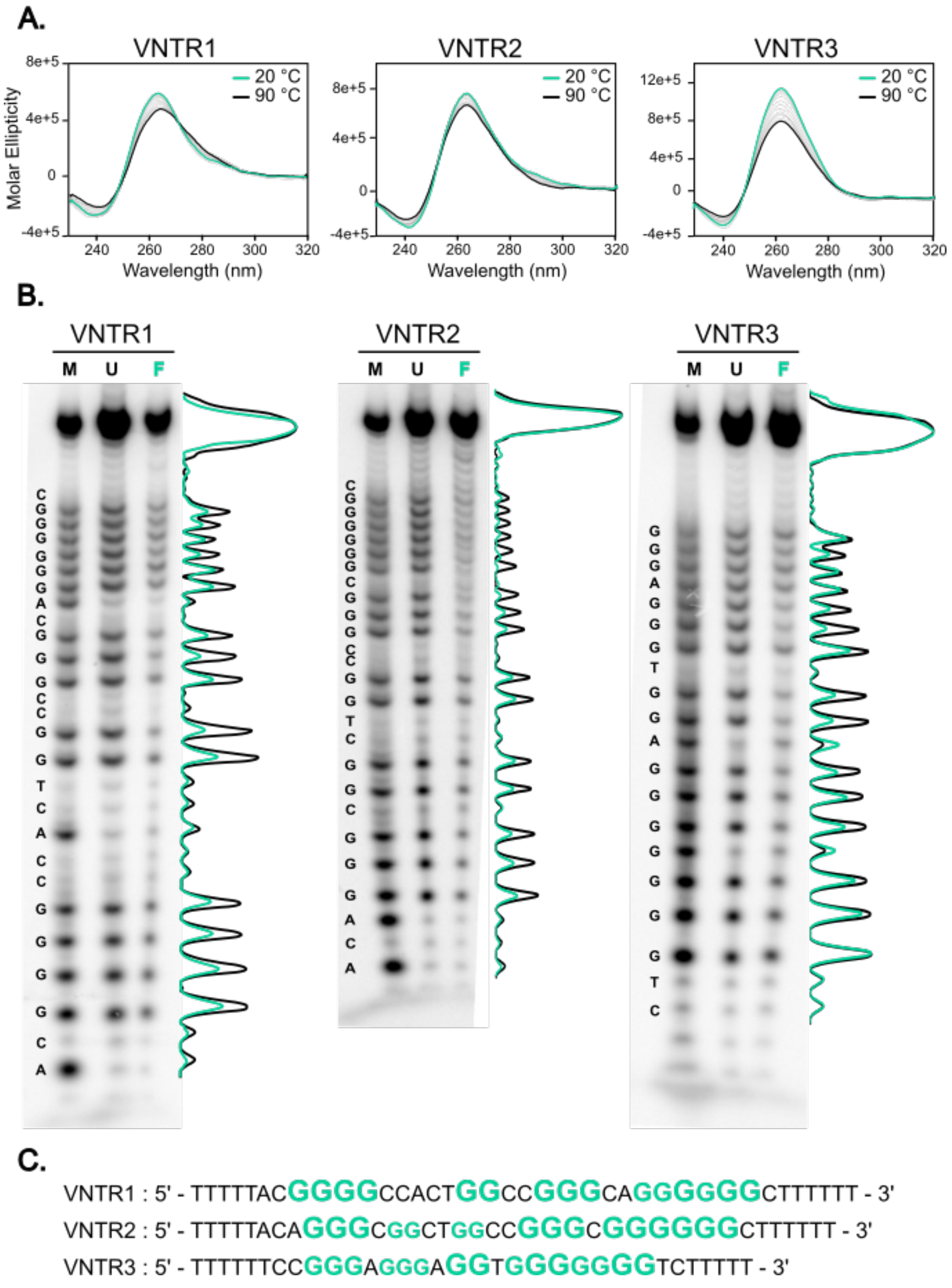

Figure S1: ***In vitro* characterization of VNTR pG4s. A.** CD melting experiments of putative G4-forming sequences found in the XDP SVA VNTR domain. All sequences tested show a G4 parallel structure at low KCl concentration (10 mM). **B.** DMS footprinting of the folded (F) and unfolded (U) VNTR1-2-3 sequences. M stands for sequence marker for adenines and guanines. The black and green profiles indicate the degree of protection of each guanine identified in the unfolded and folded state respectively. **C.** The VNTR sequences as analyzed by DMS footprinting. Bases protected by the DMS alkylation/cleavage are shown in green. The font size is proportional to the degree of protection.

Figure S2

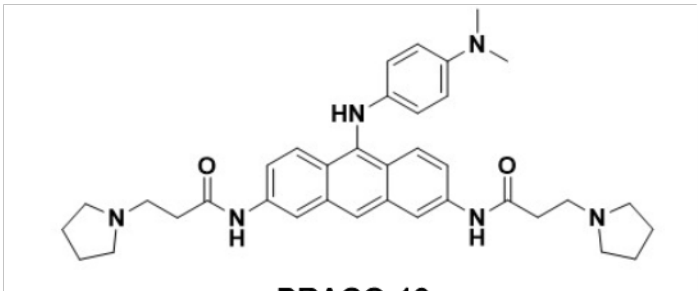

BRACO-19

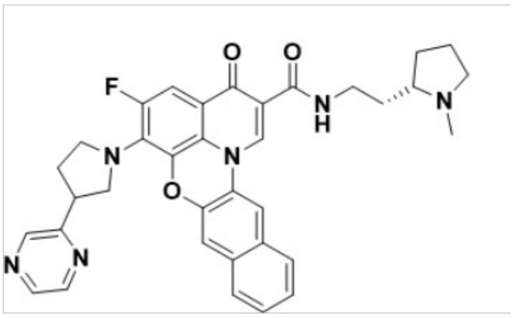

Quarfloxin

Figure S2: Molecular structure of the G4 ligands used in this study. BRACO-19 (B19) on the left, Quarfloxin (Q) on the right.

Figure S3

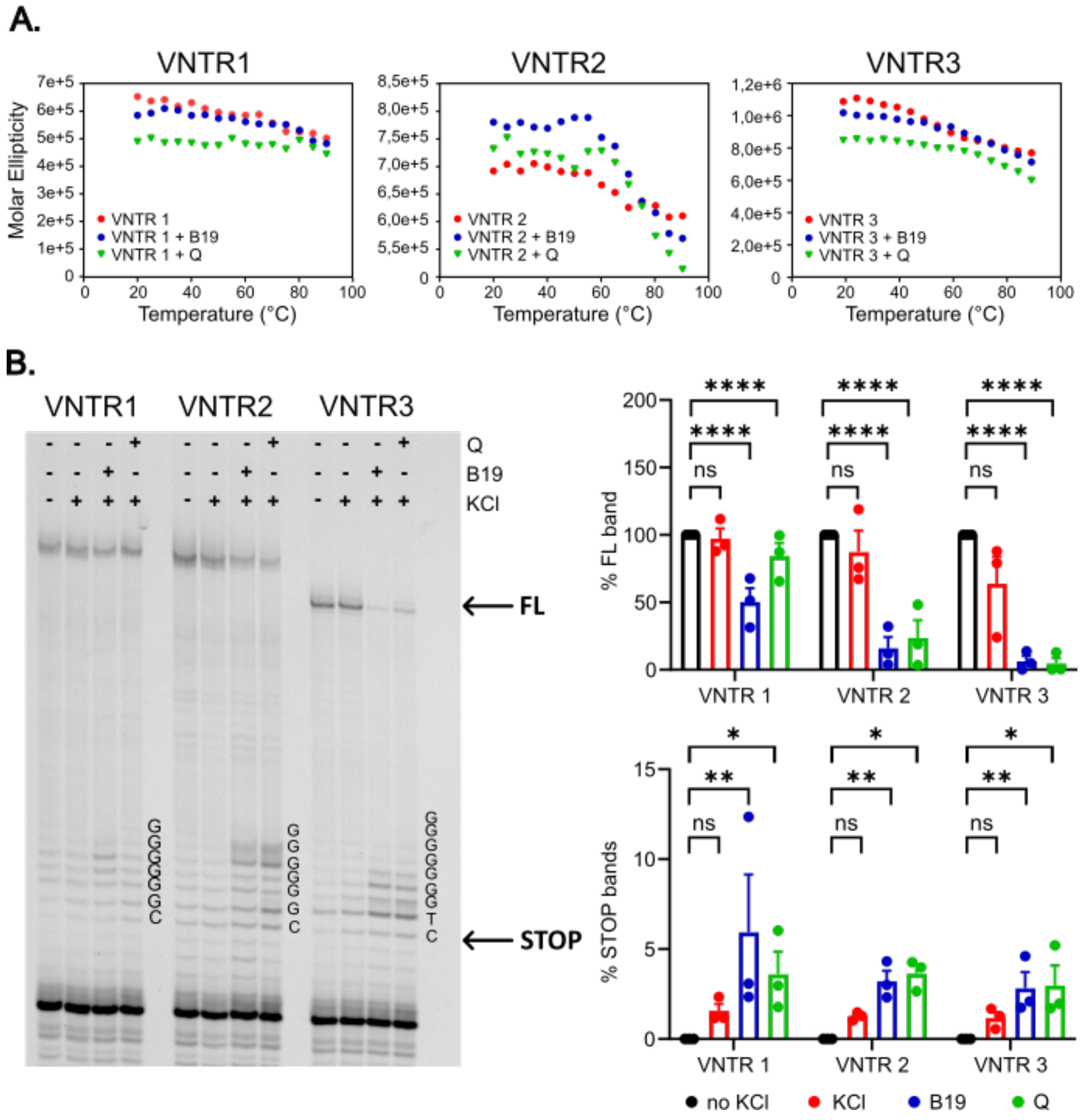

Figure S3: VNTR pG4s are stabilized by G4 ligands. A. The CD melting profile of the folded sequence (in red) or in the presence of a G4 ligand (B19 in blue; Q in green). The G4 structure is stabilized in the presence of the G4 ligand. Melting temperatures are given in Table S2. **B.** Taq pol stop assay of the VNTR G4s sequences. The assay was performed under different conditions, such as in the presence of KCl 100 mM and G4 ligand to stabilize the G4 sequence. Under these conditions, the full-length product is reduced and stop bands appear, especially in the presence of G4 ligands. Statistical analysis was performed on three independent experiments using ANOVA test with multiple comparisons setting the condition without KCl as reference.

Figure S4

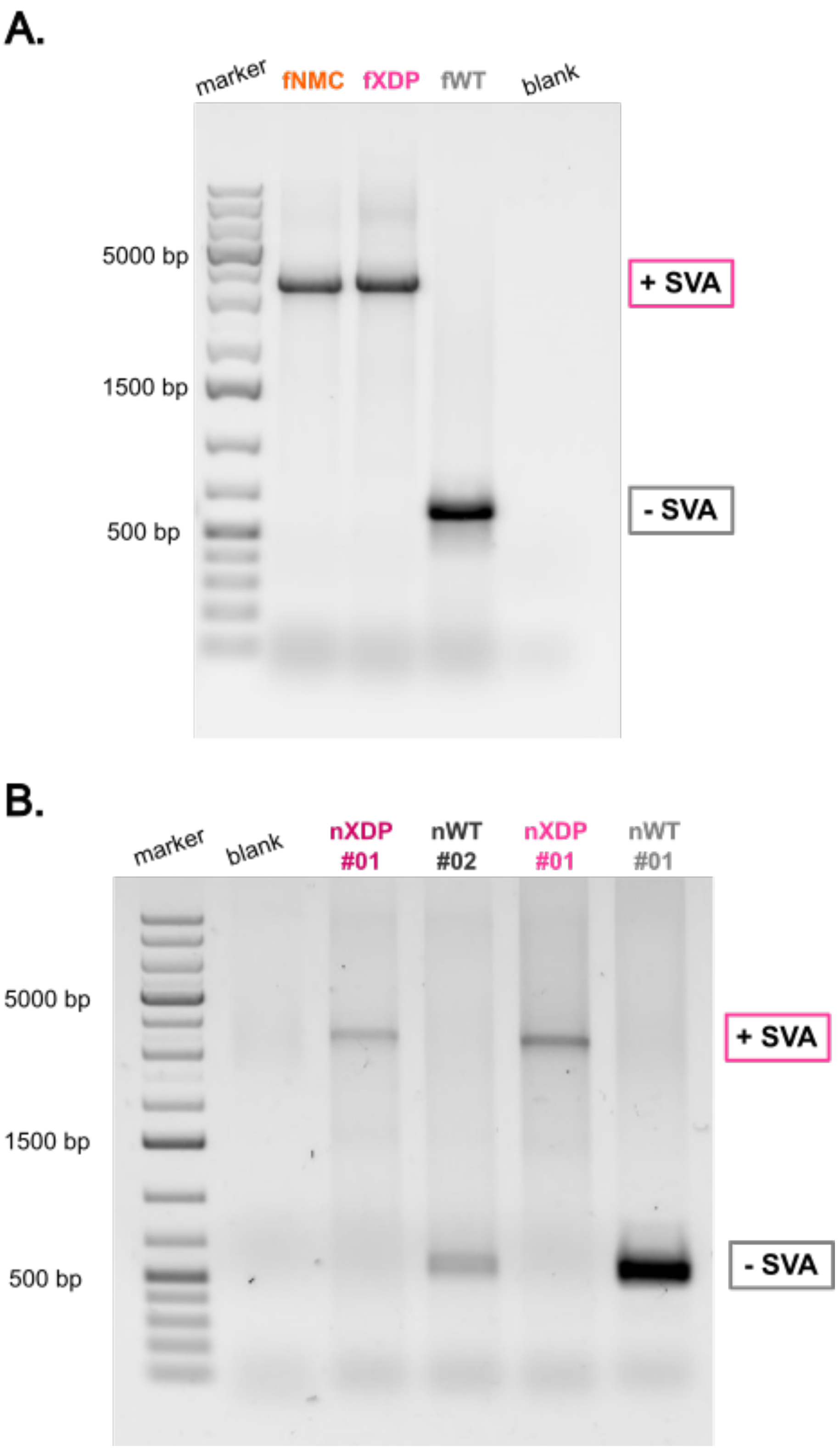

Figure S4: Genotyping of the hFibs cell lines (A) and NPCs (B) used in this study. The cell lines are indicated at the top of the gel. The PCR primers are flanking the SVA insertion, therefore XDP and NMC patients show a shifted band (about 3200 bp) compared to the WT (600 bp) due to the presence of the XDP SVA retrotransposon (2600 bp).

Figure S5

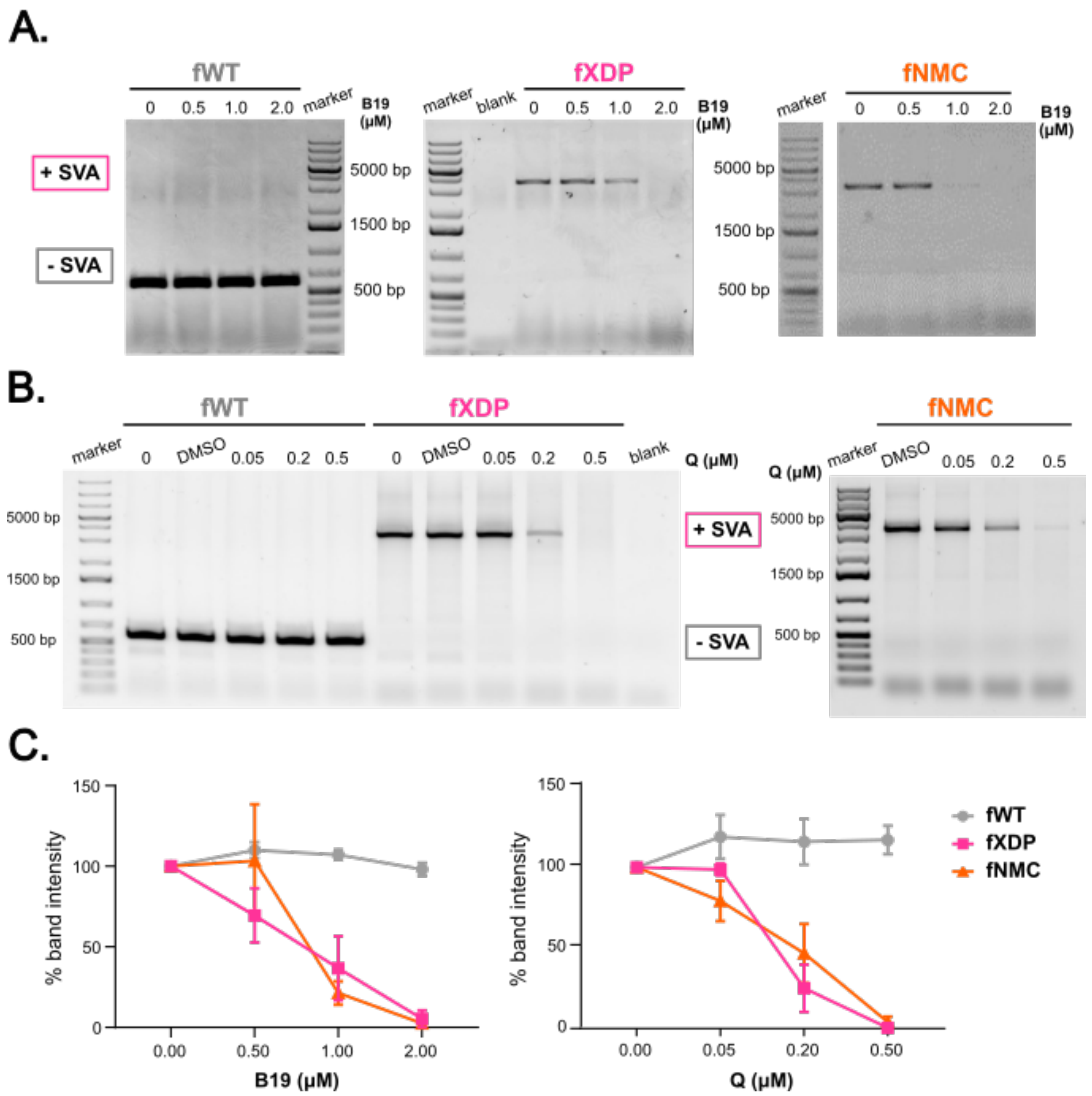

Figure S5: PCR STOP assay in the presence of different G4 ligands. A-B. Agarose gel of PCR stop assay with increasing concentration of G4 ligand. B19 (A) or Q (B) on fWT,fXDP and fNMC.fWT templates. **C.** Gel band quantification of the PCR stop assay in the presence of G4 ligand. fXDP, fNMC and fWTgenomic DNA. For the two SVA carrier cell lines fXDP and fNMC, SVA amplification was reduced in the presence of B19 (left panel) or Q (right panel).

Figure S6

>**chrX:71440295+71440888** (594bp)

AAACATTTACTCATTGAAGGACATTCAGATTTTCACTATTTTGCCATTGTGAGTAAAGCTGCTGTGAGCAA
TCATAGACAGGGTTTTGGATGAGTTTAATTTTTCATTTCTTTGGGATAAATGCCCAAGAATAGAATGGTAT
ATGTTTAGTTTTACAAGACACGGCACTATTTCATTTTTTTTTTTTCCACATCAGATGTGGAAAAAAAATGTA
CTGGCATTGTAATAAGGCTTGAGGGAGGCACATCTCACACATGAACGTGAAAACCCAATGTCGTCACAC
TTACGTCATCATAAGCTTATGAACTACAAAAAGATTGTGCCACACTTTTGGAGTGTCTGTACCATTTTACA
TTCCCACCAGCAATGTATGTGATCCAGTTTCTCTGCAGCATTCACTGTTACCACTTTTTAAAATTTTAGCT
GTTCTTCTATTGTGATTTTACTTGGCATTTTTCTTTTTTTAAATTAAAAACATTTTCTTGCTCTCGGTCTTAA
AATTATTTCCTCAAATACATTGTGTTTTT

pG4 found by QGRS = 0

Figure S6: **QGRS pG4 prediction on WT PCR amplicon.** The PCR amplicon obtained using primers flanking the XDP SVA insertion in the ctrl cell lacks pG4

Figure S7

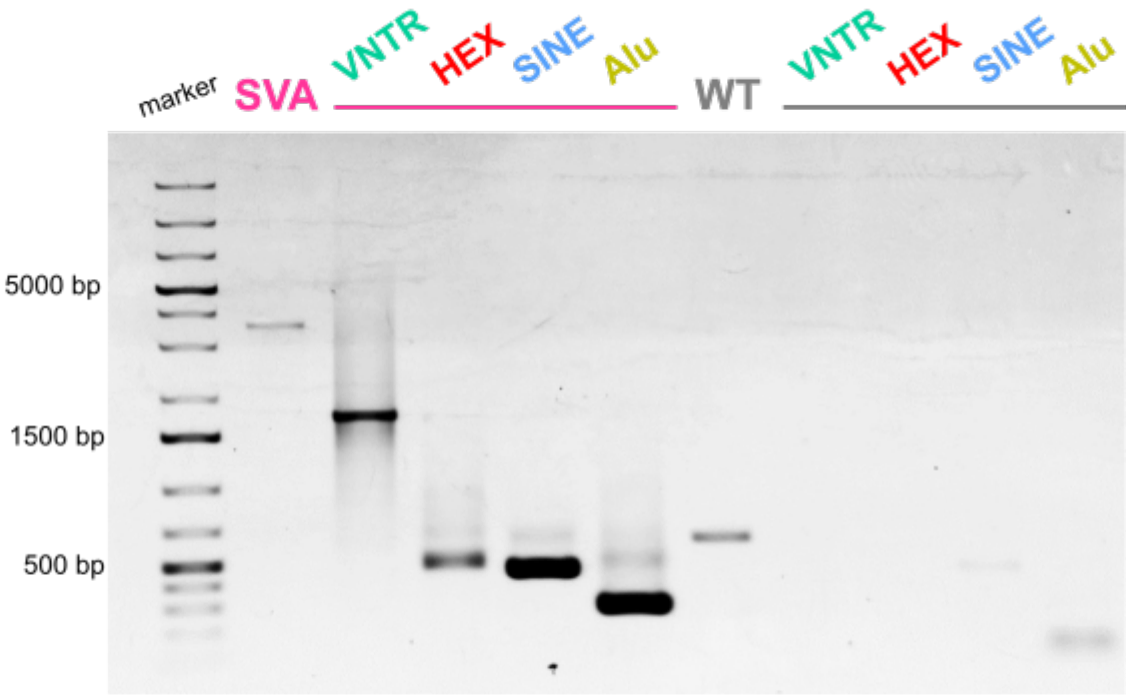

Figure S7: Amplification of SVA domains by nested PCR. PCR amplification of each domain of the XDP SVA using WT or SVA gel purified bands as template. The VNTR domain band is 1691 bp, HEX is 500 bp (depending on the number of repeats in the patient), SINE and Alu are 444 bp and 276 bp respectively. As expected, no amplification of any of the domains is obtained when the WT band is used as template.

Figure S8

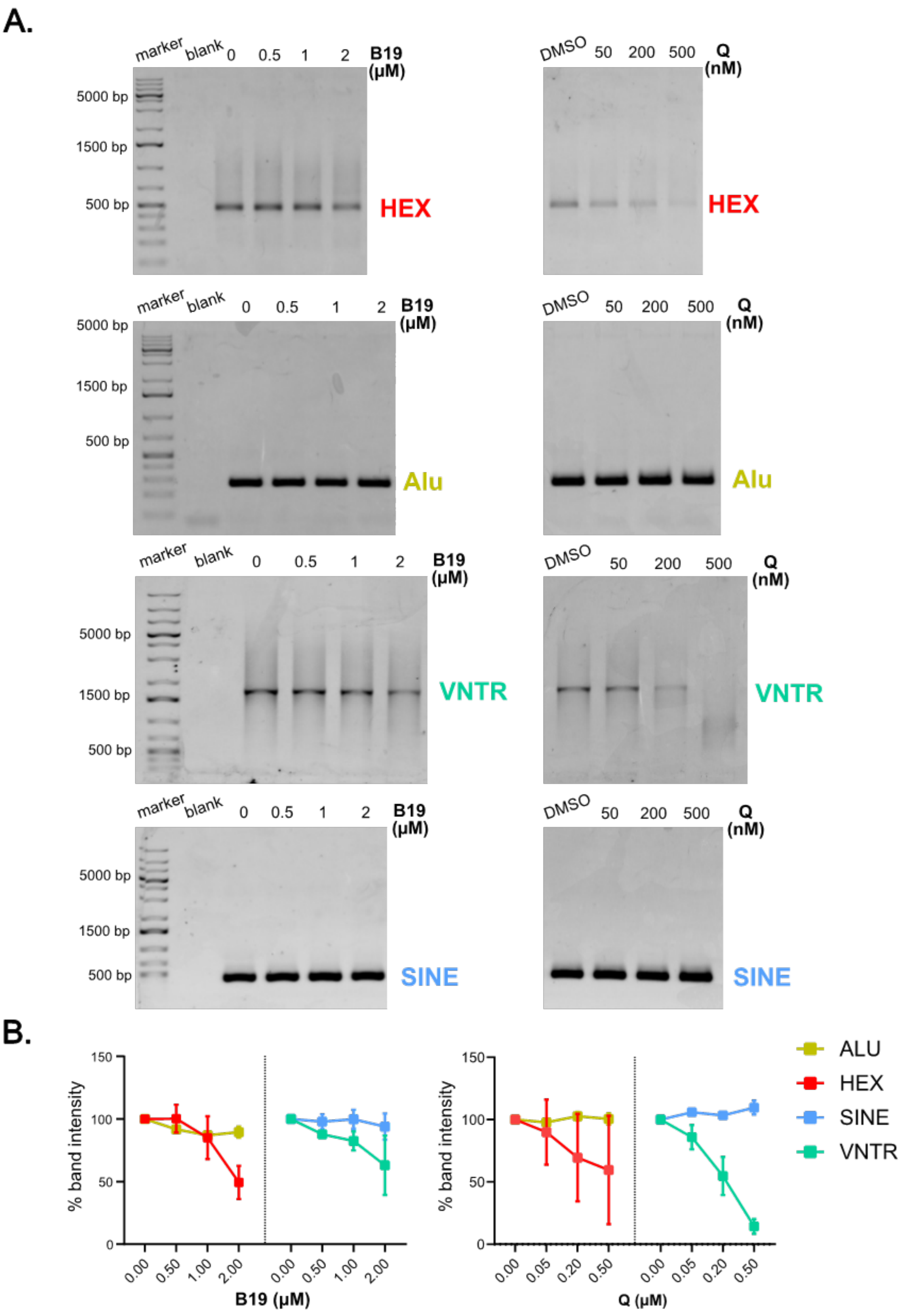

Figure S8: Nested PCR stop assay in the presence of different G4 ligands. A. Agarose gel of nested PCR stop assay in the presence of increasing concentration of B19 (left panel) or Q (right panel). **B.** Gel band quantification of the nested PCR stop shown in **A.** HEX (in red) and VNTR (in green) amplification was reduced in the presence of B19 (left panel) or Q (right panel) in a concentration-dependent manner, whereas SINE (in blue) and Alu (in yellow) amplification was not affected.

Figure S9

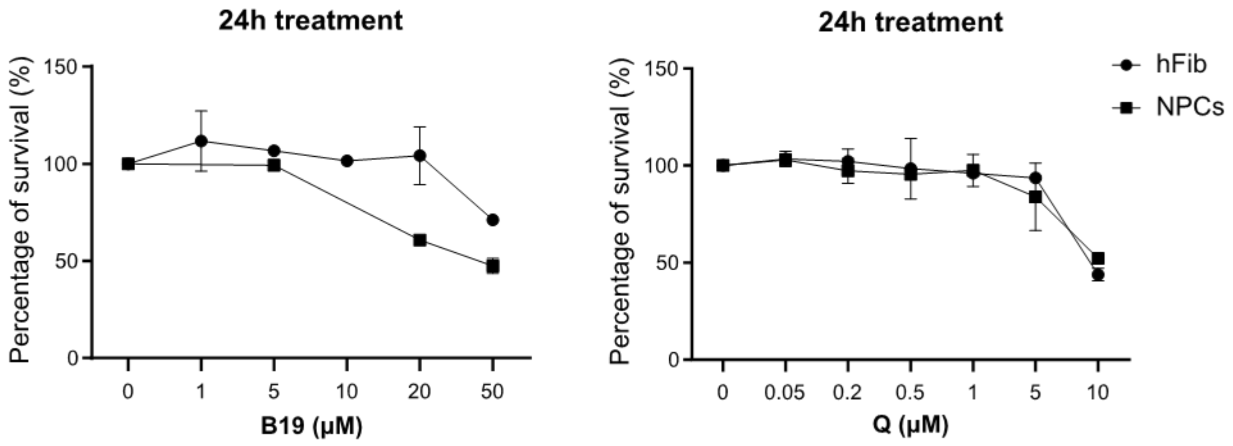

Figure S9: Cytotoxicity curve of G4 ligands in XDP hFibs and NPCs after 24 h treatment. NPCs are more sensitive to B19 treatment than hFibs. Q shows similar cytotoxicity for both cell lines.

Figure S10

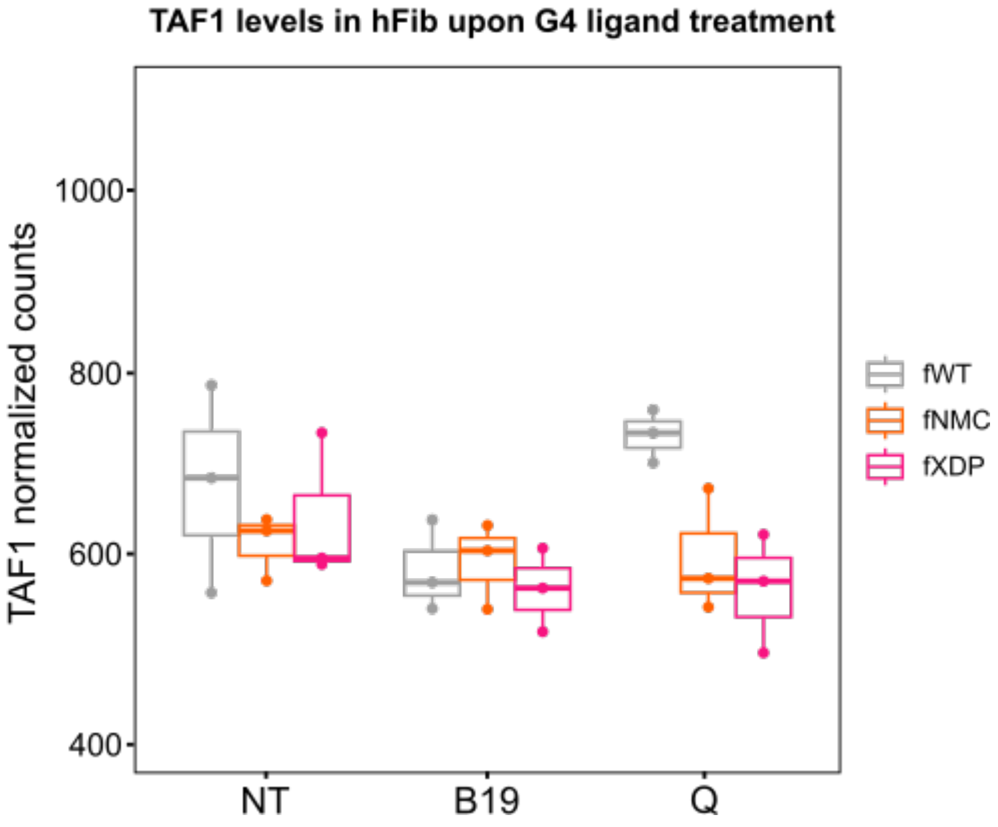

Figure S10: *TAF1* full-length transcript levels in hFib cells after 24 h treatment with B19 and Q. *TAF1* transcript levels in hFib untreated (NT) or treated with G4 ligands B19 and Q (n = 3) from control (fWT), non-manifesting carrier (fNMC) and fXDP patients' cells

Figure S11

A.

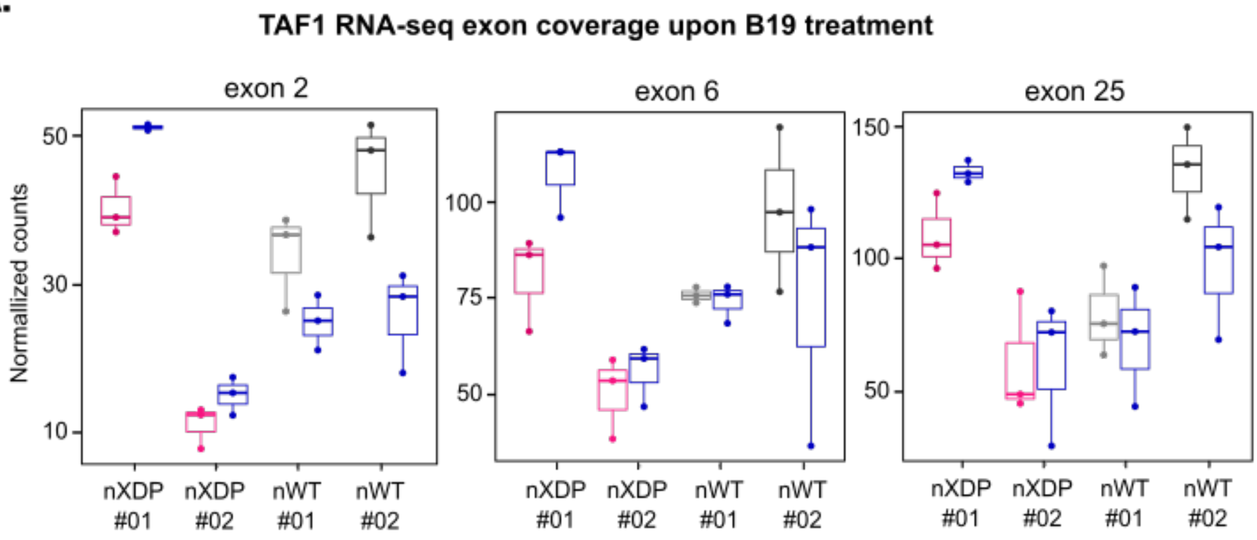

B.

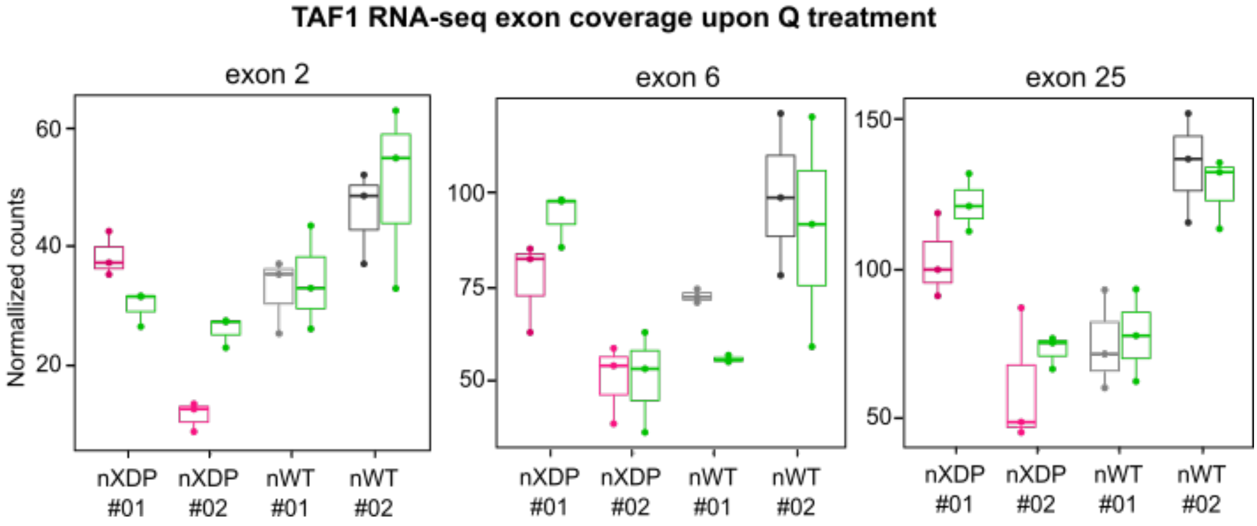

Figure S11: RNA-seq TAF1 exon before SVA insertion levels in NPCs upon B19 (in blue) or Q treatment (in green). In nXDP cells in two out of three exons tested G4 ligand treatment increase exon levels. On the other hand, in nWT cells there is an opposite trend.

Figure S12

A.

Differential expression analyses of alternative splicing events in nXDP #01

upon B19 treatment **upon Q treatment**

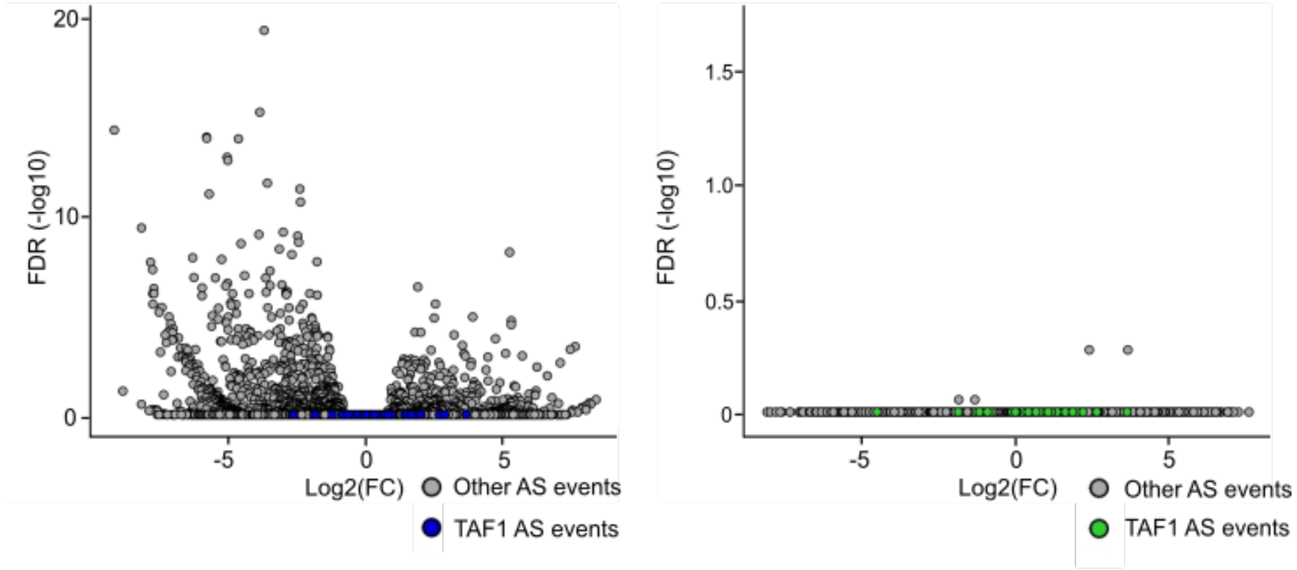

B.

Differential expression analyses of alternative splicing events in nXDP #02

upon B19 treatment **upon Q treatment**

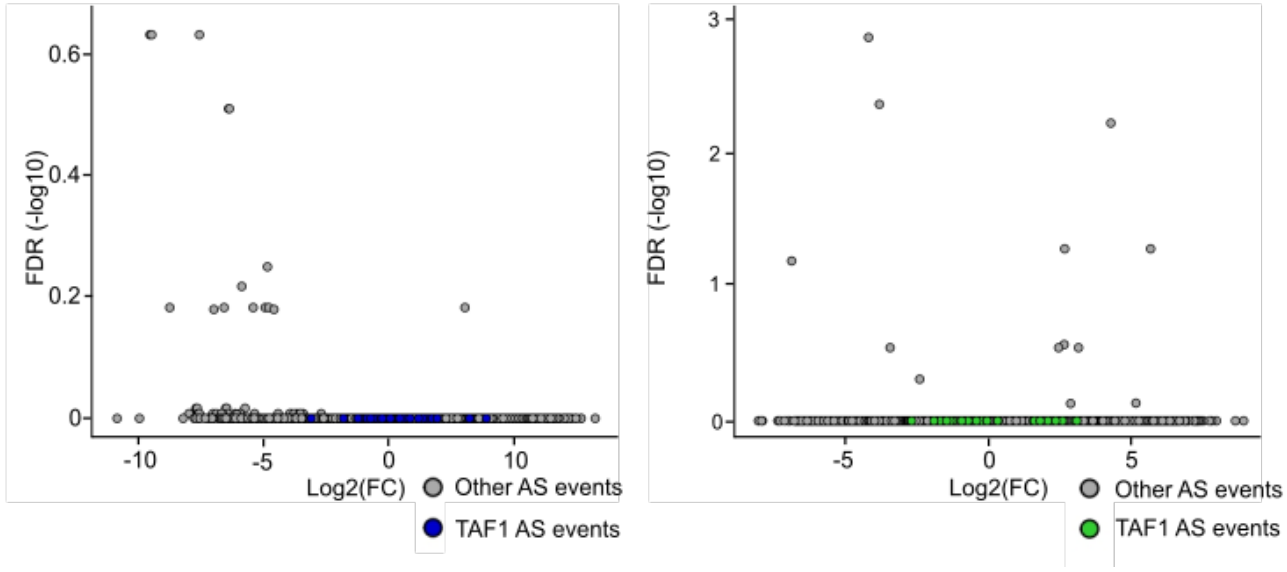

Figure S12

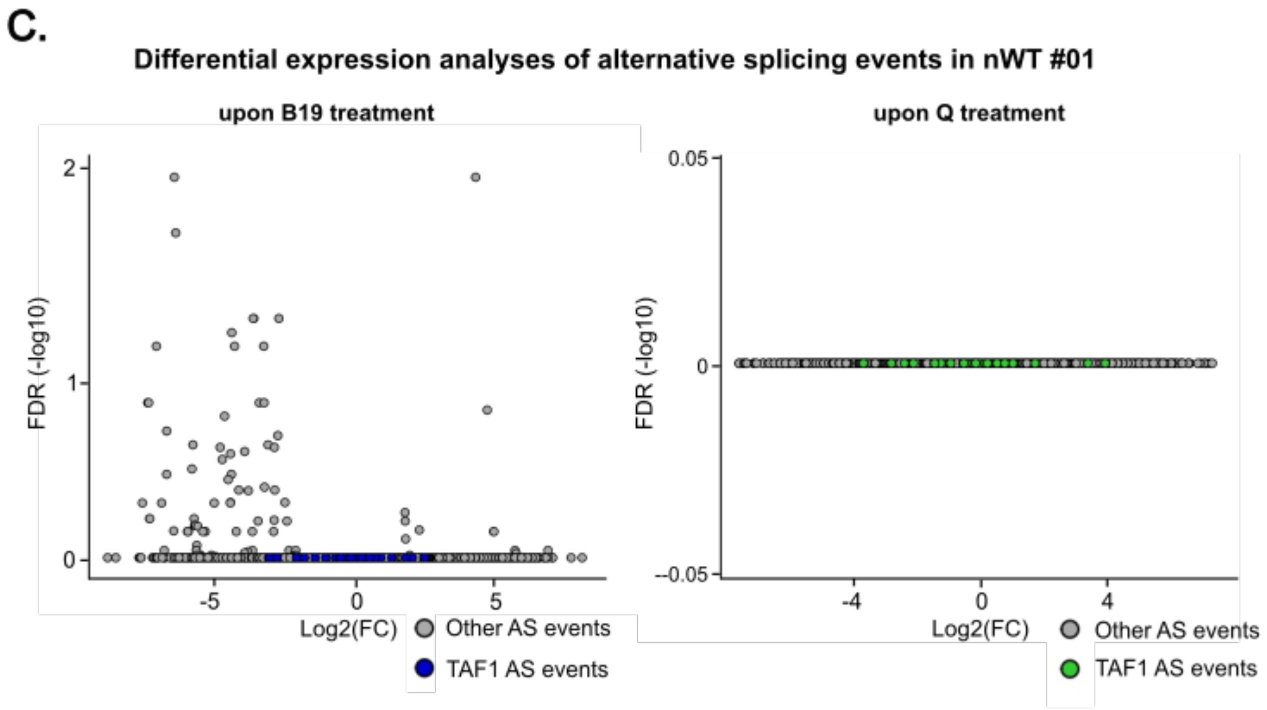

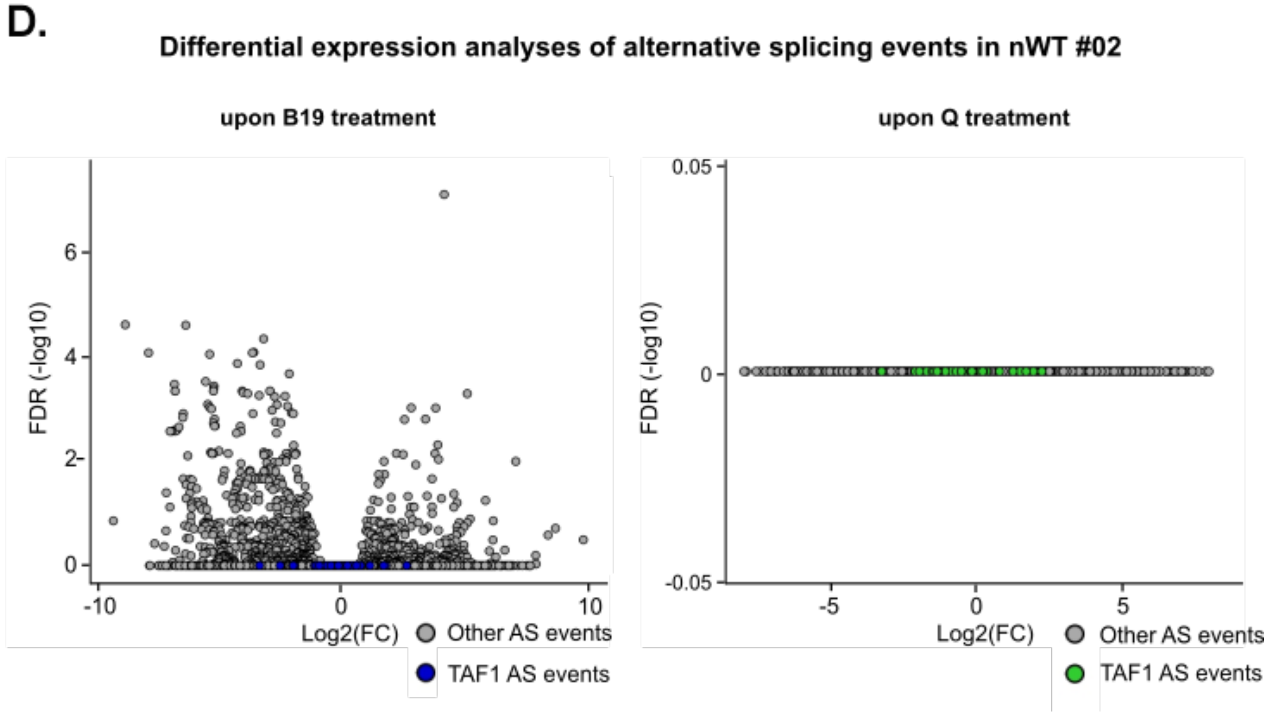

Figure S12: TAF1 alternative splicing events in XDP (A-B) and WT (C-D) NPCs treated with G4 ligands. Volcano plots from RNA-seq data showing the alternative splicing events in all genes upon treatment with B19 (left panels) and Q (right panels). The events at the TAF1 gene upon treatment with B19 are shown in blue and upon treatment with Q in green. As shown, no alternative splicing events are observed in TAF1 upon G4 ligand treatment in both nXDP and nWT cell lines.

Figure S13

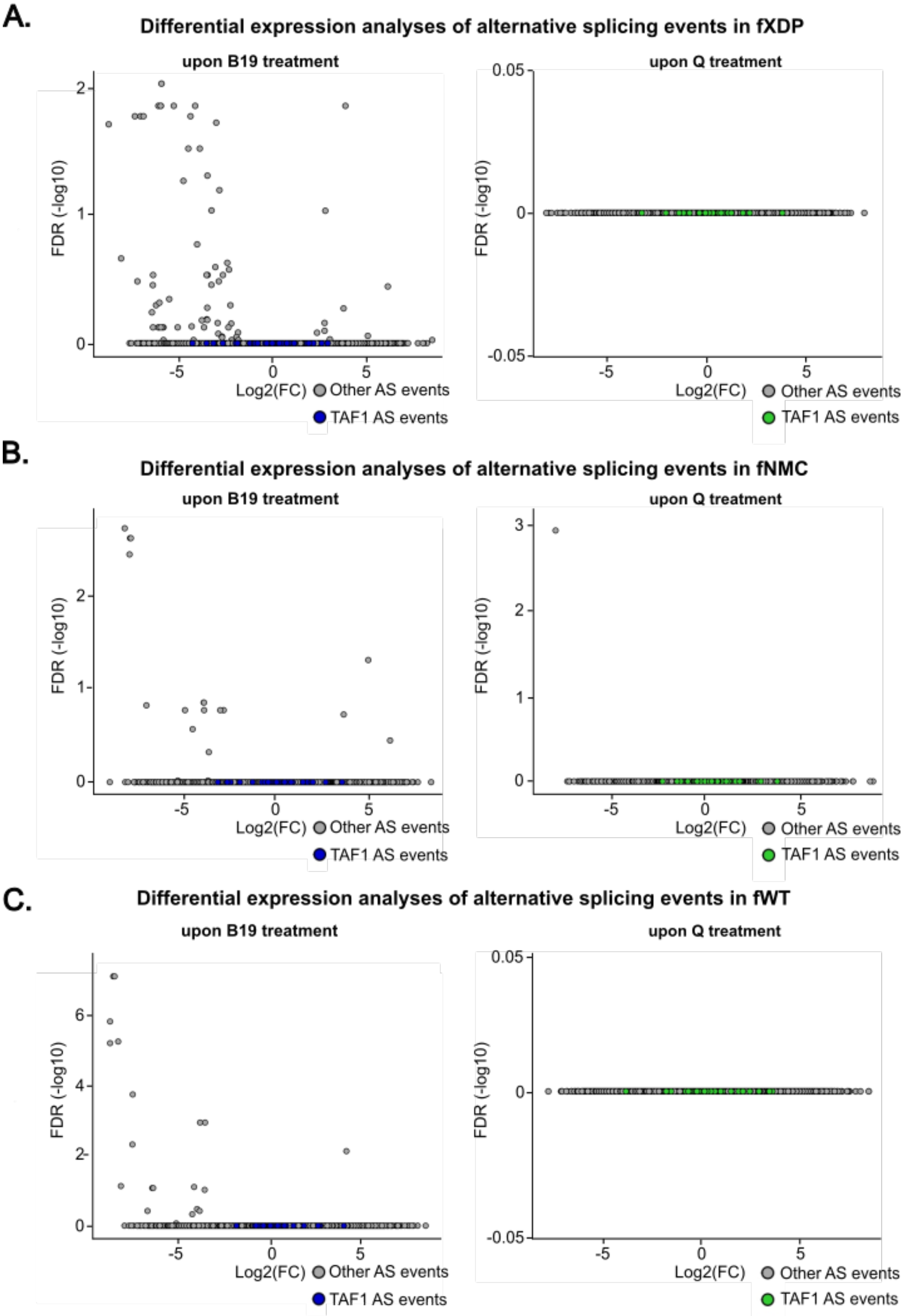

Figure S13: TAF1 alternative splicing events in XDP (A), NMC (B) and WT (C) hFibs treated with G4 ligands. Volcano plots from RNA-seq data showing the alternative splicing events in all genes upon treatment with B19 (left panels) and Q (right panels). The events at the TAF1 gene upon treatment with B19 are shown in blue and upon treatment with Q in green. As shown, no alternative splicing events are observed in TAF1.

Figure S14

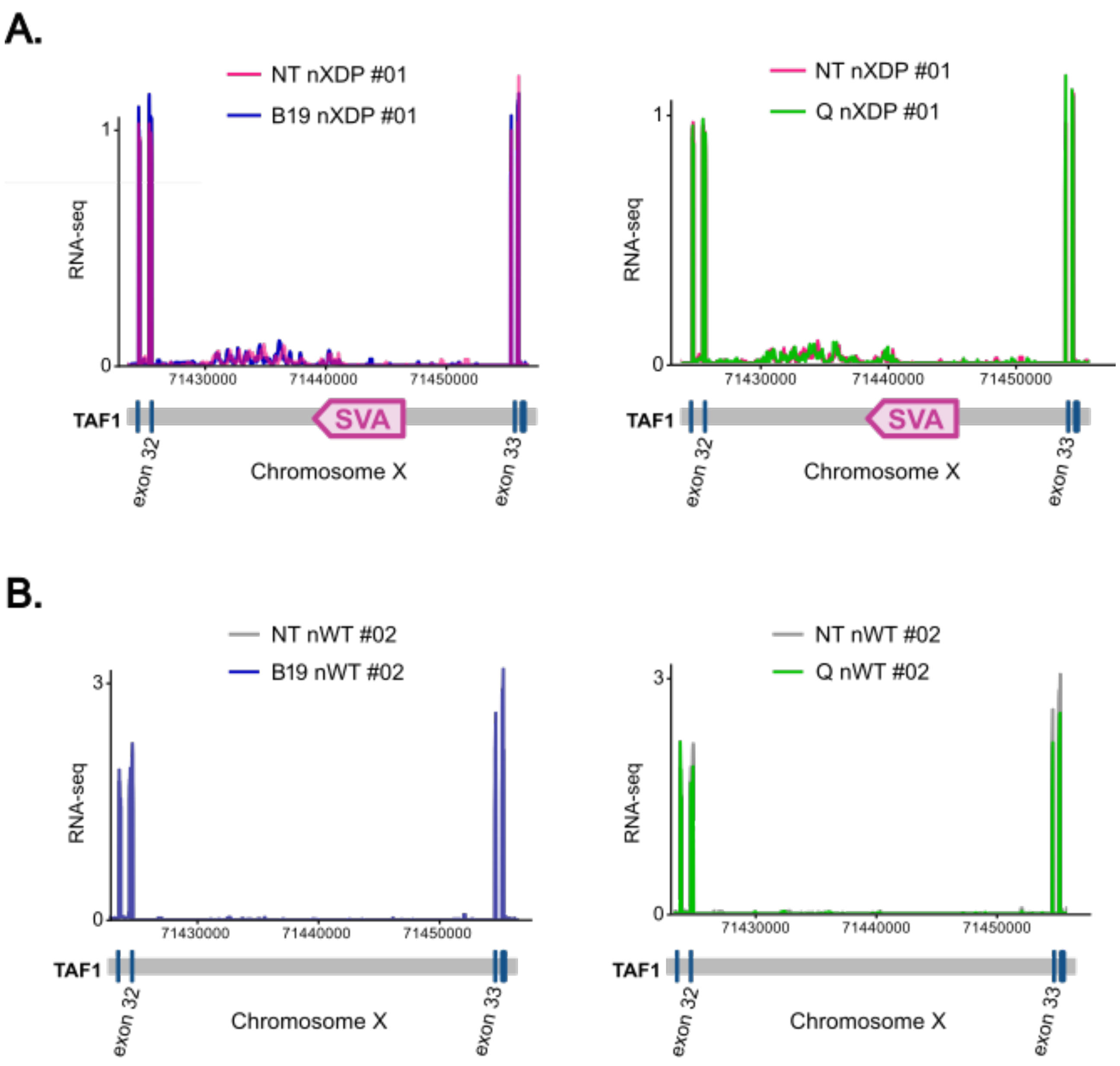

Figure S14: *TAF1 intron retention levels in nXDP #01 (A) or nWT #02 (B) NPCs cells.* RNA-seq coverage of TAF1 intron 32 after B19 (blue) or Q (green) treatment.

Figure S15

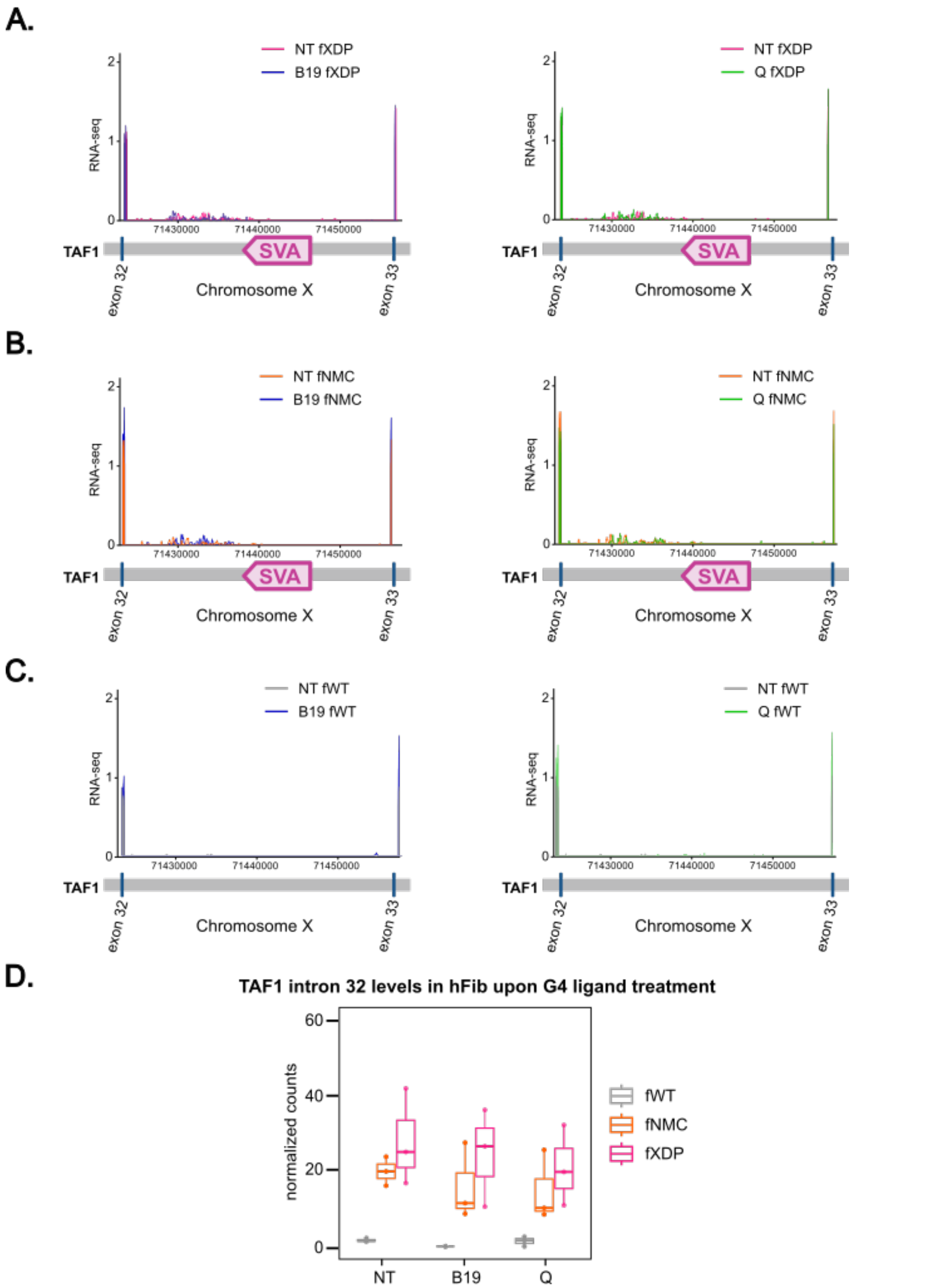

Figure S15: ***TAF1 intron retention levels in hFib cells.*** RNA-seq coverage of TAF1 after B19 (blue) or Q (green) treatment on intron 32 in fXDP (**A**), fNMC (**B**) and fWT (**C**). **D**. Box plot of intron 32 in hFib upon G4 ligand treatment.

Figure S16

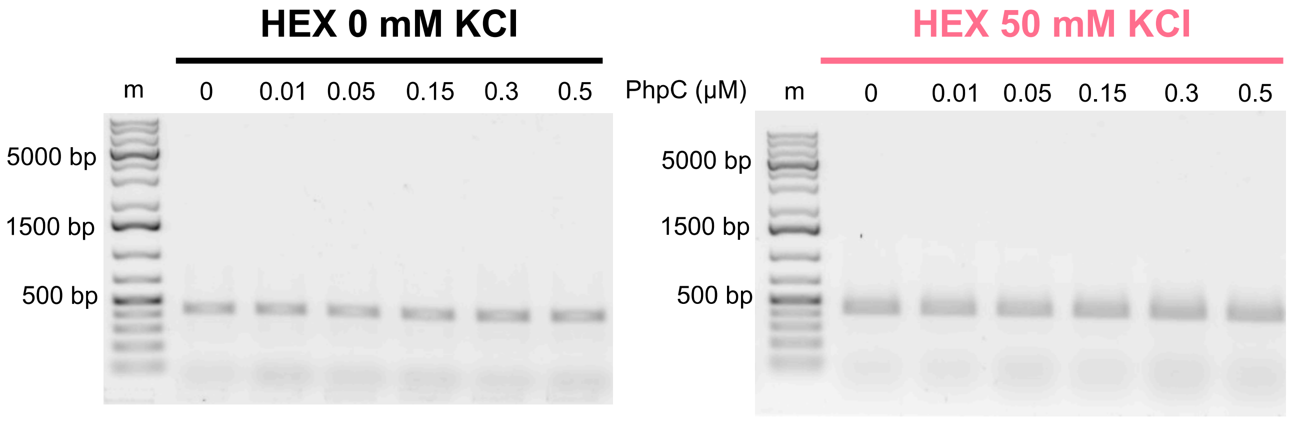

HEX 0 mM KCl
HEX 50 mM KCl
PhpC (μM)
m
0
0.01
0.05
0.15
0.3
0.5
5000 bp
1500 bp
500 bp

Figure S16: Nested PCR stop assay of the HEX domain in the presence of increasing concentrations of PhpC. Agarose gel of nested PCR stop assay with SVA folded without (left panel) or with 50 mM KCl (right panel) as starting condition. No changes in HEX amplification are observed under these conditions.

Figure S17

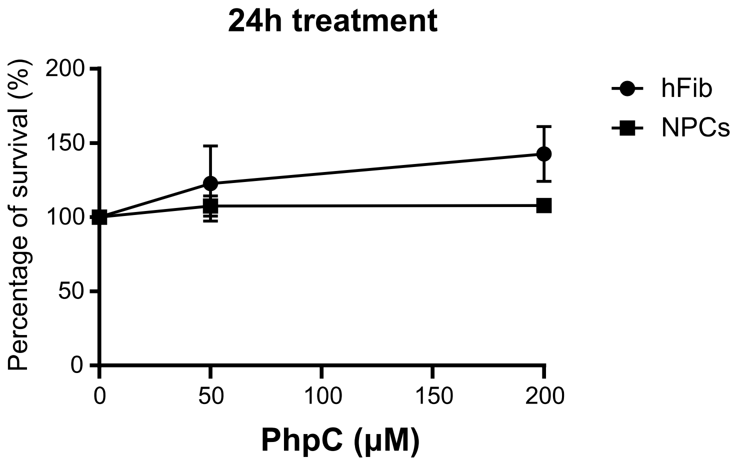

Figure S17: Cytotoxicity curve of the G4 destabiliser PhpC in XDP hFib and NPCs after 24 hours treatment. The compound is non-cytotoxic even at the highest concentrations tested.
